# Generative AI Factors, Cognitive Engagement, and Patient Safety Learning in Medical Education: Mixed Methods Study

**DOI:** 10.2196/100828

**Published:** 2026-10-27

**Authors:** Gilbert Golahi, Ferdi Antonio, Devina Nur Annisa

**Affiliations:** 1 Department of Hospital Administration Graduate School of Management Pelita Harapan University South Jakarta, Jakarta Indonesia; 2 Siloam Hospitals TB Simatupang South Jakarta, Jakarta Indonesia; 3 JEC Eye Hospitals & Clinics West Jakarta, Jakarta Indonesia

**Keywords:** patient safety competencies, generative artificial intelligence, medical clerkship education, self-efficacy, human-AI interaction

## Abstract

**Background:**

Generative AI (GenAI) is increasingly used during medical training, including clerkships in which learning takes place alongside supervised patient care. Its educational value may depend on how students interpret, verify, and regulate AI-generated output.

**Objective:**

This study explored medical clerkship students’ accounts of GenAI use and examined how GenAI-related factors were associated with self-reported ward-based infection-prevention practice through self-efficacy in AI-generated usage and perceived AI self-control balance.

**Methods:**

We used an exploratory sequential mixed methods design. Eight medical clerkship students with experience using GenAI participated in semistructured interviews analyzed using reflexive thematic analysis. The qualitative findings were considered alongside theory and prior evidence to specify a theory-based model. This model was subsequently examined through an online cross-sectional survey of 214 eligible students recruited from 5 private universities in the Jakarta Metropolitan Area, Indonesia. Quantitative associations were analyzed using partial least squares structural equation modeling with bootstrapping.

**Results:**

The interviews yielded 16 themes concerning appraisal of AI-generated output, confidence in using GenAI, interaction control, patient-safety awareness, and infection-prevention learning. The structural paths were unchanged, although 3 low-loading measurement indicators were removed before hypothesis testing. Global model fit was limited (estimated-model standardized root mean square residual 0.089; normed fit index 0.691), and pairwise residual fit was not used as a retention criterion. Explainability quality exposure and perceived algorithmic fairness were positively associated with both self-efficacy and perceived AI self-control balance. Trust calibration literacy was associated with self-efficacy but not perceived AI self-control balance. Privacy concern was positively associated with self-efficacy (β=0.294, *P*<.001), contrary to the hypothesized direction, but not with perceived AI self-control balance. Self-efficacy (β=0.477, *P*<.001) and perceived AI self-control balance (β=0.407, *P*<.001) were positively associated with self-reported ward-based infection-prevention practice; the model explained 51.9% of its variance. Specific indirect effects through self-efficacy were observed for all 4 antecedents and through perceived AI self-control balance only for explainability and fairness. A small positive interaction was observed between promotion of patient safety and self-efficacy (β=0.122, *P*=.04); the corresponding interaction involving perceived AI self-control balance was not observed.

**Conclusions:**

Self-efficacy was the more consistent pathway linking students’ appraisals of GenAI with self-reported ward-based infection-prevention practice. By integrating students’ accounts with quantitative testing of a theory-based model, this study extends research that has largely focused on GenAI acceptance and general educational use by examining a safety-oriented outcome within clinical training. The findings add evidence on the human factors through which GenAI use may relate to patient-safety learning, although the cross-sectional, self-reported data do not establish causal or clinical effects. Medical schools and teaching hospitals could use these findings to inform clerkship guidance on supervised verification of AI-generated output, patient-data protection, alignment with local protocols, and continued human responsibility for clinical decisions.

## Introduction

Generative AI (GenAI) is increasingly embedded in medical education, changing how students seek explanations, organize information, practice reasoning, and manage cognitive effort during demanding learning tasks [1,2]. In medical clerkship education, this development is particularly important because learning occurs in safety-critical clinical environments where students’ documentation, communication, and clinical reasoning are closely connected to the development of patient safety competencies [3,4]. Recent evidence indicates that medical learners already use GenAI for study support, clinical learning, and academic work, while also expressing concerns about accuracy, bias, and overreliance [4-6]. These concerns are relevant because GenAI may support reflective learning when used appropriately, but may also become a convenient answer source if learners do not remain cognitively engaged [2,4,7].

The educational value of GenAI is not automatic. Its contribution depends on task design, AI literacy, supervision, and the extent to which students remain active in interpreting and verifying AI-generated outputs [2,4,7,8]. This issue is especially salient in clerkship settings, where learners participate in patient-facing routines under supervision and gradually develop professional judgment through workplace-based learning [3,4]. Although GenAI has been discussed as a tool for feedback, clinical reasoning support, documentation, and learning-resource development, many institutions are still developing formal policies and training structures to guide its use in medical education [2,8,9]. This gap creates a need to understand how students use, experience, and regulate GenAI during clinical learning.

Patient safety is a central dimension of health care quality and concerns the prevention of avoidable harm during the delivery of care [10,11]. Health care–associated infections (HAIs), or nosocomial infections, remain a major patient safety challenge despite advances in infection prevention and control practices [11,12]. Systematic reviews and meta-analyses suggest that the burden of HAIs is particularly significant in low- and middle-income countries, where health system capacity, infection-control resources, and implementation consistency may vary across settings [13,14]. AI-supported systems are also increasingly being explored for HAI surveillance, outbreak monitoring, risk prediction, and infection-prevention monitoring, illustrating the growing role of digital tools in safety-critical hospital functions [12,15].

However, the potential contribution of AI to infection prevention does not depend on algorithms alone. Prior work emphasizes that human factors influence how AI outputs are interpreted, trusted, questioned, and translated into practice [15]. This is highly relevant for medical clerkship students, who occupy a transitional position between formal education and supervised clinical participation. Clerkship students contribute to ward routines such as documentation, communication, patient preparation, and infection-prevention behaviors, even though they remain under supervision [3]. As many of these students are familiar with digital technologies and increasingly exposed to GenAI tools, they represent an important learner group for understanding how AI-supported learning may relate to patient safety–oriented practice [6,16-19].

In this context, critical thinking involves questioning AI-generated output, comparing it with clinical evidence and supervisor guidance, and deciding whether the output should be accepted, revised, or rejected. Earlier studies on health technology adoption have often been informed by the technology acceptance model (TAM), which explains technology use through perceived usefulness, perceived ease of use, attitudes, and behavioral intention [20]. The TAM remains useful for examining adoption, including when AI systems are customized or embedded in nonstandard workflows; however, it does not by itself explain safe GenAI use in clinical learning environments, where patient safety, professional accountability, and supervision are central concerns [21-23]. GenAI systems can generate fluent and plausible outputs even when the content is incomplete or incorrect, creating risks of automation bias, misplaced confidence, and uncritical acceptance of AI-generated suggestions [22,23]. Medical education research therefore requires models that move beyond acceptance and examine the human factors that shape how learners engage with AI responsibly.

Existing studies have examined GenAI use in medical education, including students’ perceptions, learning engagement, academic use, and institutional preparedness [2,4-6,9,17,18]. However, less is known about how GenAI-related factors are associated with patient-safety learning in clerkship settings, particularly in relation to ward-based infection-prevention practice. Much of the literature still emphasizes acceptance, satisfaction, intention to use, or general educational utility, with less attention to how students’ confidence, perceived control, and risk awareness may relate to safety-oriented learning in clinical environments [2,4,21]. This distinction is relevant because clerkship students learn through supervised participation in clinical work, where the use of GenAI may intersect with documentation, infection-prevention routines, and patient care.

To address this gap, the study focused on medical clerkship students as learners positioned between classroom-based education and supervised clinical practice. It linked students’ perceptions of GenAI with 2 human-centered mechanisms, which are self-efficacy in AI-generated usage and perceived AI self-control balance (PAISCB), and examined self-reported ward-based infection-prevention practice as a safety-oriented learning outcome. After the qualitative phase was completed, the themes were considered alongside existing theory to develop the conceptual framework and directional hypotheses. This work was completed before quantitative data collection and analysis. The themes informed the selection and arrangement of theory-based constructs but were not used to create new psychometric constructs or questionnaire items. Consistent with social cognitive theory, the resulting paths were treated as connected parts of a single model [24].

The 12 directional hypotheses were organized into 3 related groups. H1-H8 concerned the associations between 4 appraisals of AI-generated output and the 2 human-centered mechanisms. H9 and H10 concerned the associations of these mechanisms with self-reported ward-based infection-prevention practice. H11 and H12 examined whether promotion of patient safety moderated the 2 mechanism-outcome associations. The individual paths were retained because they represented distinct theoretical relationships, but the findings were interpreted within these 3 groups rather than as unrelated hypothesis tests.

The first group considered how students’ appraisals of AI-generated output might relate to their confidence and perceived control when using GenAI. Perceived algorithmic fairness may influence whether students regard AI-generated output as legitimate and appropriate for use in supervised learning [25,26]. Trust calibration literacy is relevant because students need to determine when AI output can be relied upon, when it should be questioned, and how it compares with clinical reasoning and supervisor guidance [27-29]. Explainability quality exposure may support learning by allowing students to understand the reasoning behind an output instead of accepting a recommendation at face value [30-32]. Privacy concern toward AI monitoring may have the opposite effect if concern about data use, surveillance, or unintended disclosure discourages students from engaging confidently with GenAI [33,34].

These appraisals were examined in relation to 2 aspects of student agency. Self-efficacy in AI-generated usage refers to students’ belief in their ability to use AI effectively for learning and clinical tasks [35,36]. PAISCB concerns whether students feel able to retain autonomy and control over how they initiate, guide, and end an interaction with GenAI [37,38]. Fairness, trust calibration, and explainability were expected to be positively associated with both mechanisms. Privacy concern was expected to be negatively associated with them. This reasoning informed H1-H8:

- H1: Perceived algorithmic fairness is positively associated with self-efficacy in AI-generated usage.
- H2: Perceived algorithmic fairness is positively associated with PAISCB.
- H3: Trust calibration literacy is positively associated with self-efficacy in AI-generated usage.
- H4: Trust calibration literacy is positively associated with PAISCB.
- H5: Explainability quality exposure is positively associated with self-efficacy in AI-generated usage.
- H6: Explainability quality exposure is positively associated with PAISCB.
- H7: Privacy concern toward AI monitoring is negatively associated with self-efficacy in AI-generated usage.
- H8: Privacy concern toward AI monitoring is negatively associated with PAISCB.

The second group addressed whether the 2 human-centered mechanisms were associated with ward-based infection-prevention practice. Social cognitive theory proposes that contextual conditions influence action partly through people’s beliefs about their own capabilities [24]. In clerkship settings, students apply what they learn through repeated ward activities, including documentation, communication, and infection-prevention behavior [3,39]. Confidence in using GenAI may help students use its output as a provisional learning aid, while perceived control over the interaction may allow them to decide when and how that output should inform their work. Neither mechanism implies that clinical judgment can be delegated to the system. AI-supported learning remains dependent on verification, supervision, and attention to the risks of inappropriate trust or loss of human authority [22,23,40]. Ward-based infection-prevention practice was selected as the outcome because it represents a safety-oriented aspect of clinical learning that is directly relevant to clerkship activities [41]. On this basis, H9 and H10 concerned the 2 mechanism-outcome paths:

- H9: Self-efficacy in AI-generated usage is positively associated with ward-based infection-prevention practice.
- H10: PAISCB is positively associated with ward-based infection-prevention practice.

The third group examined whether patient-safety orientation influenced the strength of these relationships. Students who place greater emphasis on patient safety may be more likely to translate confidence and control during GenAI use into cautious, verified practice. Promotion of patient safety was therefore examined as a moderator of the associations between the two human-centered mechanisms and ward-based infection-prevention practice [42]. This reasoning informed H11 and H12:

- H11: Promotion of patient safety positively moderates the association between self-efficacy in AI-generated usage and ward-based infection-prevention practice.
- H12: Promotion of patient safety positively moderates the association between PAISCB and ward-based infection-prevention practice.

The conceptual model and its component paths are presented in Multimedia Appendix 1.

This study aimed to explore how medical clerkship students described their use of GenAI in clinical learning and to examine, in a subsequent quantitative phase, the associations proposed in the qualitatively informed, theory-based model. The model linked students’ appraisals of AI-generated output and 2 human-centered mechanisms with self-reported ward-based infection-prevention practice.

The study addressed 3 research questions. First, what GenAI-related factors did medical clerkship students identify as relevant to clinical learning and patient-safety practice? Second, to what extent were the associations specified in the quantitative model observed in relation to self-reported ward-based infection-prevention practice through self-efficacy in AI-generated usage and PAISCB? Third, how did the qualitative themes and quantitative findings converge, expand upon, or diverge from one another in explaining GenAI-supported patient-safety learning?

## Methods

### Research Design Overview

This study used a sequential exploratory mixed methods design comprising an initial qualitative phase followed by a cross-sectional quantitative phase. In this study, exploratory refers to the sequence and purpose of the inquiry rather than to the development of new psychometric constructs or questionnaire items. The qualitative phase used semistructured interviews and reflexive thematic analysis to examine how medical clerkship students described GenAI use in clinical learning, including their experiences of trust, fairness, explainability, privacy, human control, and patient safety–related practice. The quantitative phase subsequently examined the associations among the corresponding theory-based constructs, self-efficacy in AI-generated usage, PAISCB, promotion of patient safety, and self-reported ward-based infection-prevention practice [43-45].

This design was selected because GenAI use in clerkship education involves both context-dependent experiences and relationships that can be examined quantitatively. The qualitative phase provided an account of how students understood and used GenAI within their clerkship setting, whereas the quantitative phase examined whether the specified relationships were observed in a larger sample. Reporting of the qualitative and mixed methods components was informed by the relevant Journal Article Reporting Standards (JARS) recommendations [46]. Construct measurement remained grounded in the source instruments described in the “Measures and Covariates” section.

Integration followed a sequential building strategy with a theory-informed abductive logic. After the qualitative themes had been developed inductively, they were considered alongside existing theory and prior evidence to develop the conceptual framework and directional hypotheses for the quantitative phase. This work was completed before quantitative data collection and analysis. The themes informed the selection and arrangement of theory-based constructs within the structural model, while the direction of the proposed relationships was supported by theory and previous empirical findings [47,48]. The quantitative phase subsequently examined the resulting model, and findings from both phases were considered together during interpretation [45,49].

### Study Setting and Recruitment Period

This study was conducted among medical clerkship students from 5 private universities in the Jakarta Metropolitan Area, Indonesia. Data collection was conducted sequentially. Qualitative interviews took place from January to February 2026, followed by recruitment for the online quantitative survey from February to March 2026. The qualitative participants represented major clinical departments, including internal medicine, surgery, pediatrics, and obstetrics, whereas the quantitative survey was distributed through the faculties of medicine of the participating universities.

### Ethical Considerations

The study was approved by the Pelita Harapan University Institutional Ethics Committee (approval number 060/MARS/EC/XII/2025). Participation in both phases was voluntary. Written informed consent was obtained before the qualitative interviews, while quantitative respondents provided electronic informed consent before beginning the survey. Before providing consent, participants were informed about the study purpose and procedures, potential risks and benefits, confidentiality safeguards, and their right to withdraw without penalty. No directly identifying information was collected through the quantitative survey. Qualitative interview transcripts were deidentified, and pseudonyms were used in place of participants’ names. Electronic study data were stored on encrypted drives and used only for academic research purposes. Participants received no financial or material compensation. No identifiable participant information is presented in this paper or supplementary materials.

### Phase 1: Qualitative Methods

The first phase used semistructured, in-depth interviews to examine students’ accounts of GenAI use in clinical and academic learning. The transcripts were analyzed using reflexive thematic analysis, with themes developed inductively before mixed methods integration [43,50,51].

#### Study Participants or Data Sources

##### Researcher Description

The qualitative phase was conducted by a 3-member research team with prior experience in qualitative and mixed methods research. GG, a medical doctor and graduate student in hospital administration at the time of the study, conducted all interviews. He had received training in qualitative research methods and led the coding and initial development of themes. FA provided methodological supervision and contributed to validation of the analysis, while DNA supported data curation and validation.

The research team recognized that GG’s clinical background and interest in GenAI could shape both the interview process and the interpretation of participants’ accounts. Contextual observations were documented in field notes, and preliminary interpretations were discussed with FA and DNA and checked against the interview transcripts before the themes were finalized. These discussions were used to examine whether the interpretations remained grounded in participants’ accounts rather than reflecting the research team’s initial expectations.

##### Participants or Other Data Sources

Qualitative data were obtained from 8 active medical clerkship students who had experience using GenAI for clinical or academic learning. Their ages ranged from 22 to 27 years; 6 were women and 2 were men. The participants represented 4 major clinical departments: internal medicine, surgery, pediatrics, and obstetrics. Each participant contributed one semistructured interview, and the thematic analysis included all 8 verbatim transcripts.

##### Researcher-Participant Relationship

The interviewer (GG) had no prior relationship with any of the participants and did not serve as their lecturer, clinical supervisor, assessor, or academic administrator. Information about the study was distributed through faculty representatives. Before the interviews, participants were informed that GG was a medical doctor and graduate student in hospital administration conducting the research. They were also told that participation was voluntary and that neither their decision to participate nor their responses would affect their academic grades, clinical rotations, or relationship with the faculty.

#### Participant Recruitment

##### Recruitment Process

Recruitment took place from January to February 2026. Faculty representatives at the participating universities distributed information about the study to medical clerkship students but were not involved in determining eligibility. With the students’ permission, the contact details of those who expressed interest were forwarded to GG, who conducted the eligibility screening and provided further information about the interview. Eleven students were approached. Three declined without providing a reason, while 8 met the eligibility criteria, provided written informed consent, and completed the interviews. The participants were recruited from several of the participating universities. No financial or material compensation was offered.

##### Participant Selection

Participants were selected purposively based on their relevance to the qualitative aim. Eligible participants were active medical clerkship students with direct experience using GenAI for clinical or academic learning. Selection also sought variation across major clinical departments, including internal medicine, surgery, pediatrics, and obstetrics. Sample adequacy was assessed using the concept of information power, considering the focused study aim, the specificity of the participant group, the relevance of their experience, the use of an established theoretical framework, and the depth of the interviews [52]. After 8 interviews, the dataset was judged to provide sufficient contextual range and depth to address the purpose of the qualitative phase, and recruitment was closed. The later interviews largely reinforced the patterns already identified and did not add substantively different contextual insights.

#### Data Collection

##### Interview Procedures

Qualitative data were collected from January to February 2026 through individual, face-to-face, semistructured interviews conducted in Indonesian by GG. Each participant completed one interview in a private consultation room. Only the participant and the interviewer were present. The interviews lasted between 45 and 60 minutes, with a mean duration of 53.8 (SD 4.7) minutes. No repeat interviews were conducted.

The interview guide was developed from the study objectives, relevant theory and literature, and discussions within the research team. FA and DNA reviewed the guide before data collection began. The interviews covered participants’ use of GenAI in clinical and academic learning, their interpretation of AI-generated outputs, and their experiences related to trust, fairness, explainability, privacy, human control, and patient safety–related practice. The order of questions was adapted to the direction of each interview, and follow-up questions were used when clarification or further detail was needed. Field notes were made during and immediately after each interview. The same interview guide was used throughout the qualitative phase, with no substantive changes made during data collection. The interview guide is available from the corresponding author upon reasonable request.

##### Recording and Data Transformation

All interviews were audio-recorded using a password-protected digital recording device. GG transcribed the recordings manually and verbatim in Indonesian. Each transcript was checked in full against the corresponding audio recording, and any transcription errors were corrected before analysis. Names and other potentially identifying details were removed, and each participant was assigned a pseudonym.

The original Indonesian transcripts were used for coding and thematic analysis. After the analysis, quotations selected for publication were translated into English by GG and reviewed by DNA to ensure that their meaning and context were retained. Any differences in interpretation were resolved through discussion. Full transcripts were not returned to participants; participant review was limited to thematic summaries and is described in the “Methodological Integrity” section. Audio recordings, deidentified transcripts, and field notes were stored on encrypted drives accessible only to the research team.

#### Analysis

##### Data-Analytic Strategy

The interview transcripts were examined using Braun and Clarke’s 6-phase reflexive thematic analysis [50,51]. This analysis was intended to develop a contextual account of how clerkship students experienced and interpreted GenAI use in clinical learning. GG conducted coding and theme development manually without dedicated qualitative data-analysis software. GG read each transcript several times before coding began. The full transcript served as the primary unit of analysis, with codes applied to meaningful segments of text. Initial codes were developed across the dataset without a predetermined coding framework. The theory-based constructs included in the quantitative model were not used as coding categories at this stage.

Codes were compared within and across interviews to identify recurring patterns as well as differences in participants’ accounts. Related codes were brought together into candidate themes, which were then reviewed against the coded extracts and the full dataset. Themes were combined, separated, or refined when their boundaries were unclear or their supporting data were insufficiently coherent. The central idea and scope of each theme were subsequently defined before the final names were assigned. These phases were recursive rather than strictly sequential, allowing movement between transcripts, codes, and candidate themes throughout the analysis. No separate formal discrepant-case analysis was conducted; however, contrasting accounts were retained when they clarified variation within a theme or qualified a broader pattern.

Theme development remained inductive and data-driven [50,51]. After the themes had been finalized, they were considered alongside existing theory during the abductive integration described in the “Mixed Methods Analysis” section. The direction and theoretical rationale of the proposed relationships were established from prior theory and empirical evidence before quantitative data collection and analysis.

##### Methodological Integrity

Credibility and confirmability were supported through comparison across participants, peer debriefing, and participant feedback. During theme development, GG compared coded extracts within and across transcripts to determine whether candidate themes were adequately supported while retaining variation in participants’ accounts. Developing codes and themes were discussed with FA and DNA through peer debriefing. These discussions were used to question assumptions, consider alternative readings of the data, and refine the scope of the themes rather than to establish intercoder agreement.

Three participants reviewed concise summaries of the themes and commented on whether the summaries reasonably reflected the substance of their accounts. This process involved thematic summaries rather than the return or correction of full transcripts. Final themes were checked against the source transcripts and presented with deidentified participant quotations to show how the interpretations were grounded in the interview data.

The a priori constructs were not used as initial coding categories. No formal discrepant-case analysis was undertaken, although contrasting accounts were retained when they qualified a broader pattern. These boundaries informed the subsequent mixed methods interpretation.

### Phase 2: Quantitative Methods

#### Inclusion and Exclusion

The quantitative phase included medical clerkship students aged 18 to 27 years from 5 private universities in the Jakarta Metropolitan Area. Respondents were eligible if they had completed or were currently undergoing a major clinical rotation and reported frequent or very frequent use of GenAI, including ChatGPT, Google Gemini, Grok, BLACKBOX AI, or another platform, in clinical learning. A submission was not included if the screening responses did not confirm eligibility or if the main questionnaire was incomplete.

#### Participant Characteristics

The final quantitative analytic sample comprised 214 medical clerkship students. Table 1 summarizes their sex and age distribution, frequency of GenAI use in clinical learning, and the platforms they reported using. Respondents could select more than one platform; therefore, the percentages reported for GenAI platforms do not sum to 100%.

**Table 1:** Demographic characteristics and generative AI (Gen AI) use profile of 214 medical clerkship students recruited in the quantitative phase from 5 private universities in the Jakarta Metropolitan Area, Indonesia.

Description and category		Value, n (%)
Sex		
	Male	56 (26)
	Female	158 (74)
Age (years)		
	18-24	194 (91)
	25-27	20 (9)
GenAI usage frequency in the clinical study setting		
	Frequent	162 (76)
	Very frequent	52 (24)
GenAI platforms used		
	ChatGPT	193 (90)
	DeepSeek	57 (27)
	Google Gemini	135 (63)
	Meta AI	89 (42)
	Microsoft Copilot	29 (14)
	Claude.AI	40 (19)
	Consensus	28 (13)
	Grok	21 (10)
	BLACKBOX AI	80 (37)
	Others	2 (1)

#### Sampling Procedures

The quantitative phase used purposive, nonprobability sampling. From February to March 2026, faculty representatives at the 5 participating universities distributed the study information and a link to the cloud-based survey. Their role was limited to distributing the invitation; they did not determine eligibility or have access to individual responses. Students decided independently whether to open the survey and provide electronic informed consent. Mandatory screening questions were presented before the main questionnaire.

Faculty representatives sent the survey invitation directly to medical clerkship students identified as potential participants. Eligibility was not assumed from receipt of the invitation but was determined through the mandatory screening questions presented before the main questionnaire. The quantitative participant flow is presented in Multimedia Appendix 2.

#### Sample Size, Power, and Precision

The required sample size was estimated using an a priori power analysis in G*Power 3.1. (Franz Faul, Edgar Erdfelder, Axel Buchner, and Albert-Georg Lang). Assuming a medium effect size (*f*²=0.15), statistical power of 0.80, an alpha level of .05, and 7 predictor variables, the analysis indicated that at least 103 participants were required [53]. The final analytic sample comprised 214 respondents and therefore exceeded the minimum sample size determined by the power analysis.

The calculation addressed statistical power for the structural analysis rather than precision for estimating a population prevalence. A margin-of-error calculation was not used because the study applied purposive, nonprobability sampling.

#### Measures and Covariates

The quantitative survey used a structured questionnaire consisting of closed-ended items. Four GenAI-related constructs were examined as antecedents: AI-perceived algorithmic fairness [25], AI-trust calibration literacy [54], AI-explainability quality exposure [30], and AI-privacy concern toward AI monitoring [34]. The model included self-efficacy in AI-generated usage [36] and PAISCB [38] as mediating constructs. Promotion of patient safety was examined as a moderating variable [42], while ward-based infection-prevention practice was treated as the dependent variable [41].

PAISCB referred to students’ perceived autonomy and control over their interaction with GenAI. In this construct, “balance” denotes the student’s sense of retaining control while using AI rather than a formal division of authority between the learner and the system. The measure was not intended to assess clinical oversight, accountable decision-making, or compliance with clinical governance procedures [38].

Sex, age, GenAI usage frequency, and the platforms used were collected to describe the sample but were not entered as covariates in the structural model. No additional covariates were included.

#### Data Collection

Quantitative data were collected from February to March 2026 through an individually accessed cloud-based survey. After completing the electronic consent and eligibility screening steps described above, eligible respondents proceeded to the main questionnaire. They were instructed to answer based on their own experiences of using GenAI during medical clerkship learning. Instructions were provided at the beginning of each questionnaire section, and completion typically required approximately 8 to 12 minutes. No IP addresses or other directly identifying information were collected. After data collection closed, the survey data were downloaded for response screening and statistical analysis.

#### Quality of Measurements

All questionnaire items were translated into Indonesian and reviewed by a linguistic expert for semantic accuracy, conceptual equivalence, and relevance to the study context. The adapted questionnaire was then assessed by a 5-member expert panel comprising 3 academics, 1 information technology professional, and 1 infection prevention and control professional. The panel reviewed each item for clarity, relevance, and appropriateness for medical clerkship students. Minor wording revisions were made in response to their assessment.

Before the main survey, 40 medical clerkship students were approached for a preliminary pilot and readability assessment, of whom 30 participated. They were drawn from the same target population but were not included in the main analytic sample. The assessment examined whether the instructions and item wording were understandable and appropriate to the intended clinical-learning context. The questionnaire was finalized after this assessment.

Several procedural measures were also used to limit common method bias [55]. The survey instructions stated that there were no right or wrong answers and asked respondents to answer according to their own experiences with GenAI. Items representing the antecedent, mediating, moderating, and outcome constructs were arranged to reduce obvious response patterns. These measures were procedural; no marker variable or other dedicated statistical measure of common method bias was included.

#### Instrumentation

The questionnaire comprised items adapted from previously validated instruments and adjusted to the medical clerkship and GenAI-supported learning context. No ad hoc instrument or newly written questionnaire item was introduced. The response-scale length of each source instrument was retained: AI-trust calibration literacy used a 7-point Likert scale, PAISCB used a 6-point Likert scale, and the remaining constructs used 5-point Likert scales. These response scales were not converted to a common number of categories during data collection. Apart from the planned reverse coding of the negatively worded ward-based infection-prevention items, the original response formats were retained. Construct definitions and response-scale lengths are provided in Multimedia Appendix 3, while the adapted item wording and reverse-coding status are provided in Multimedia Appendix 4.

The questionnaire used in the main survey contained 35 indicators adapted from 8 source measures. The indicators were analyzed as individual items rather than as parcels or summed scale scores. All 8 measurement models were specified as reflective, and the Likert-type responses were treated as metric indicators in the partial least squares structural equation modeling (PLS-SEM) analysis.

#### Masking

Masking did not apply to the quantitative phase because the study used a nonexperimental cross-sectional survey with no intervention, experimental manipulation, random allocation, or condition assignment. All respondents completed the same questionnaire, and the outcomes were self-reported. There were therefore no intervention providers or independent outcome assessors to be masked.

#### Psychometrics

The expert assessment yielded Aiken V values above 0.40 and item-level content validity index values above 0.80 for all questionnaire items after minor wording revisions. In the preliminary assessment involving 30 medical clerkship students, Cronbach α exceeded 0.70 and the average variance extracted (AVE) exceeded 0.50 for each construct. These findings were considered sufficient to proceed with the main survey.

The psychometric properties of the scores obtained from the main sample were examined as part of the PLS-SEM measurement model assessment [56]. Indicator reliability was evaluated using outer loadings. Indicators with loadings between 0.40 and 0.70 were considered in relation to their effect on construct reliability, convergent validity, and conceptual coverage rather than being removed solely based on the loading value. Internal consistency was examined using Cronbach α, rho_A, and composite reliability, with values of 0.70 or higher considered acceptable. Convergent validity was assessed using the AVE, with values above 0.50 indicating adequate convergence.

The initial measurement model contained 35 indicators. Three indicators from the promotion of patient safety measure had low outer loadings. They were removed after their loadings were considered together with internal consistency, convergent validity, and the conceptual coverage of the construct. The model was then reestimated with 32 indicators. Each construct retained at least 3 indicators, and the removal did not change the definition of the promotion of patient safety construct. The retained indicators and sample-specific estimates are reported in the “Measurement Model Estimates” section and in Multimedia Appendix 4.

Discriminant validity was evaluated using the heterotrait-monotrait ratio of correlations (HTMT) point estimates and bootstrapped 95% CIs. A point estimate below 0.90 was used as a general guideline, while the CIs were examined to determine whether they included 1.00. Estimates approaching 0.90 or intervals with upper limits above 0.90 were interpreted cautiously rather than treated as unequivocal evidence of construct separation [56]. The sample-specific psychometric estimates are reported in the “Measurement Model Estimates” section and in Multimedia Appendices 4-6.

#### Conditions and Designs

The quantitative phase used a nonexperimental, cross-sectional observational design. All constructs were measured once through the same online questionnaire. There was no intervention, manipulation of study variables, random allocation, comparison group, or repeated measurement. The conceptual framework and the direct, indirect, and moderating relationships were finalized after the qualitative phase and before quantitative data collection and analysis. No interim analysis or data-dependent stopping rule was used; recruitment ended at the close of the prespecified data-collection period. The estimated relationships were interpreted as cross-sectional associations and not as evidence of temporal or causal effects.

#### Data Diagnostics

Before analysis, submissions were screened for eligibility and completion of the main questionnaire. Item responses were then checked for missing values, values outside the permitted response ranges, and coding inconsistencies. Response-pattern checks were descriptive and were not used as an automatic exclusion rule; no response was removed solely because the same category had been selected across multiple items. No additional cases were excluded after the analytic dataset had been formed.

The final dataset of 214 respondents contained no item-level missing data; therefore, missing completely at random testing and data imputation were not performed. Observed ranges and item distributions were inspected before model estimation. Skewness and kurtosis were used descriptively, while standardized construct scores with an absolute value greater than 3.29 were treated as flags for review rather than automatic grounds for exclusion. No case was removed solely because of an extreme score or departure from normality. The negatively worded ward-based infection-prevention practice (NIFRDT) indicators were reverse-coded so that higher values represented better self-reported ward-based infection-prevention practice and lower agreement with noncompliant behaviors. No transformation was applied to the remaining item responses. The reverse-coded indicators are identified in Multimedia Appendix 4.

#### Analytic Strategy

##### Specification

The quantitative analysis used observed responses from 214 participants rather than simulated data. The structural model specified AI-perceived algorithmic fairness, AI-trust calibration literacy, AI-explainability quality exposure, and AI-privacy concern toward AI monitoring as antecedent constructs. Self-efficacy in AI-generated usage and PAISCB were positioned as intervening constructs, ward-based infection-prevention practice was the endogenous outcome, and promotion of patient safety moderated the 2 intervening-construct-to-outcome relationships. The hypothesized model and its directional paths are presented in Multimedia Appendix 1.

PLS-SEM was selected because the analysis combined explanatory and predictive aims and included several latent constructs, specific indirect effects, interaction effects, and importance-performance analysis [56]. The qualitatively informed, theory-based model was evaluated as specified, without fitting alternative structural models. The analysis followed a 2-stage procedure, beginning with measurement-model evaluation and continuing with structural-model assessment [56]. The criteria used to evaluate the measurement model are described in the “Psychometrics” section.

The final model contained 8 reflective constructs measured by 32 retained indicators. The indicators were questionnaire items adapted from several previously validated instruments and administered together in one questionnaire. Individual items were entered directly, without parceling or preliminary aggregation, and were treated as metric indicators. Each construct had at least 3 retained indicators. The structural model was recursive and contained no reciprocal or feedback paths.

PLS-SEM was estimated as a recursive component-based path model. Conventional covariance-based structural equation modeling degrees of freedom and identification tests were therefore not used because the model was not estimated by reproducing the sample covariance matrix [56]. All constructs were specified using reflective measurement models with distinct sets of indicators; each construct retained at least 3 indicators, and no reciprocal or feedback paths were included. These specifications allowed the measurement and structural parameters to be estimated in SmartPLS (SmartPLS GmbH).

The model included specific indirect pathways through self-efficacy in AI-generated usage and PAISCB. Direct paths from the 4 antecedent constructs to ward-based infection-prevention practice were not specified; accordingly, the indirect effects were not classified as indirect-only or complementary mediation. Moderation was specified through interaction terms between promotion of patient safety and each intervening construct. Promotion of patient safety and the corresponding intervening constructs were retained as constituent main effects in the outcome equation.

Participants were recruited from 5 universities. Because the respondent-level dataset did not retain a university identifier, institution-level clustering could not be estimated or adjusted for. The quantitative results therefore represent a pooled analysis across the participating universities.

##### Estimation

PLS-SEM was performed in SmartPLS version 4.1.1.2. Estimation used the path weighting scheme, initial weights of 1.0, a maximum of 3000 iterations, and a stop criterion of 10⁻⁷. Both unstandardized and standardized estimates were obtained for the structural paths, while standardized estimates were used for model evaluation.

The structural model was assessed using inner variance inflation factors, coefficients of determination (*R*²), and the magnitude and precision of the estimated path coefficients. Statistical inference was based on 10,000 bootstrap subsamples with bias-corrected (BC) CIs. Because the directional hypotheses were finalized before quantitative data collection and analysis, 1-tailed tests were conducted at α=.05, with 90% BC CIs. A hypothesis was considered supported only when the coefficient was in the proposed direction and met the prespecified statistical criterion. A statistically significant coefficient in the opposite direction was classified as unsupported.

The 12 directional paths were treated as related components of a single theory-informed model. They were considered in 3 groups: paths from the 4 antecedent constructs to the 2 intervening constructs, paths from the intervening constructs to ward-based infection-prevention practice, and the 2 interaction paths. No formal multiplicity adjustment was applied because each path represented a distinct theory-based association and the study conclusions were not based on whether at least one path reached statistical significance. All estimated paths were reported regardless of their direction or statistical significance. Interpretation considered the magnitude and direction of the coefficients, their CIs, and the consistency of the findings within each hypothesis group. The reported *P* values are therefore path-specific and unadjusted, and findings close to the nominal significance threshold were interpreted cautiously. Specific indirect effects, predictive assessment, and importance-performance analysis were treated as secondary analyses and were not used to determine support for H1 to H12.

Specific indirect effects were evaluated using the same BC bootstrap procedure. The interaction terms were estimated using the 2-stage approach in SmartPLS. Statistically significant interactions were examined using simple-slope plots to describe the associations at different levels of promotion of patient safety.

Out-of-sample predictive performance was assessed using Q²predict and Cross-Validated Predictive Ability Test (CVPAT) [57,58]. CVPAT compared the prediction errors of the PLS-SEM model with indicator average and linear model benchmarks at the overall-model and construct levels [57]. PLSpredict and CVPAT used 10-fold cross-validation repeated 10 times with a fixed seed. Predictive performance was examined at both the indicator and construct levels rather than from an overall statistic alone.

Importance-performance map analysis (IPMA) was conducted at the indicator level to compare total effects with performance scores [58]. Performance was rescaled from 0 to 100 using the theoretical endpoints of each instrument: 1-7 for AI-trust calibration literacy, 1-6 for PAISCB, and 1-5 for the remaining constructs. The original item responses were not converted to a common response scale for the PLS-SEM analysis; rescaling was applied only when calculating IPMA performance. Indicators with comparatively high importance and lower performance were considered possible priorities for educational improvement.

### Mixed Methods Analysis

Integration occurred at 2 stages. First, after the qualitative analysis had been completed, the inductively developed themes were examined alongside relevant theory and prior research. This process was used to determine which established constructs were relevant to the participants’ accounts and how those constructs should be arranged in the quantitative model. The conceptual framework and directional hypotheses were finalized before quantitative data collection and analysis. This between-phase process represented sequential building and was guided by theory-informed abductive reasoning [47,48].

An analytic matrix was used during this process to relate the qualitative themes to relevant theoretical concepts and previously validated measures. The themes informed the selection and arrangement of 4 GenAI-related appraisals, 2 human-centered mechanisms, promotion of patient safety as a moderator, and self-reported ward-based infection-prevention practice as the outcome. No new psychometric constructs or questionnaire items were developed from the interviews. The qualitative codes and themes were also not revised to make them conform to the resulting quantitative model.

The second stage of integration took place after the quantitative analysis. The qualitative findings and structural-model results were considered together to identify areas of convergence, contextual expansion, and divergence. Integration was conducted through narrative comparison, supported by a tabular presentation of the qualitative findings and their links with the theory-based constructs in the “Results” section. The qualitative and quantitative phases involved separate participant groups; the 8 interview participants were not included in the quantitative survey sample. Qualitative data were not converted into numerical variables, and no participant-level data were merged across phases. Integration therefore informed the interpretation of the findings but did not alter the quantitative estimates [45,49].

Because the qualitative findings contributed to the development of the quantitative framework, agreement between the two phases was not treated as independent confirmation. Participant accounts that did not align with the model, as well as quantitative paths that were unsupported or opposite to the hypothesized direction, were retained in the interpretation. No formal discrepant-case analysis was conducted. The integrated conclusions therefore reflected convergence, expansion, and divergence across the 2 phases rather than agreement alone.

### Validity, Reliability, and Methodological Integrity

Procedures supporting methodological integrity in the qualitative phase are described in the “Participant Selection” and “Methodological Integrity” sections. In brief, the analysis included comparisons within and across interviews, peer debriefing within the research team, and feedback from 3 participants on concise thematic summaries. Candidate themes were checked against the source transcripts, and contrasting accounts were retained when they qualified a broader pattern. Deidentified quotations were used in reporting to show the basis of the interpretations. These procedures were intended to keep the analysis grounded in participants’ accounts rather than to establish intercoder agreement [59].

Quantitative validity and reliability were addressed during questionnaire adaptation and again in the main sample analysis. The study used measures adapted from previously validated instruments, followed by linguistic review, expert assessment, and readability testing before the survey was administered. In the main sample, measurement quality was examined through indicator reliability, internal consistency, convergent validity, and discriminant validity. Heterotrait-monotrait ratio estimates were considered together with their bootstrapped CIs, and results close to the stated criterion were interpreted cautiously rather than treated as unequivocal evidence of construct separation [56]. The assessment procedures are described in the “Quality of Measurements” and “Psychometrics” sections, whereas the sample-specific estimates are reported in the “Results” section and Multimedia Appendices 1-7.

Mixed methods legitimacy was considered in relation to the sequence of the study and the role of each phase. Qualitative themes were developed inductively before they were considered alongside theory and prior evidence. The conceptual framework and directional hypotheses were then finalized before quantitative data collection and analysis. The qualitative findings informed the selection and arrangement of established constructs but were not used to create new psychometric constructs or questionnaire items. After the quantitative analysis, findings from the 2 phases were considered for convergence, contextual expansion, and divergence [45,49]. Because the qualitative findings contributed to the development of the quantitative framework, agreement across phases was interpreted as coherence rather than independent confirmation. Contrasting qualitative accounts and quantitative paths that were unsupported or opposite to the proposed direction were retained in the interpretation. The integrated conclusions were therefore limited to what could reasonably be inferred from the qualitative accounts, the cross-sectional quantitative associations, and their points of intersection.

## Results

### Phase 1: Qualitative Findings

Analysis of the 8 interview transcripts yielded 16 themes. Four domains described how students appraised AI-generated output: perceived algorithmic fairness, trust calibration literacy, explainability quality exposure, and privacy concern toward AI monitoring. The remaining themes concerned students’ confidence in using GenAI, their perceived control over the interaction, patient-safety awareness, and ward-based infection-prevention practice. Table 2 presents the themes, representative quotations, analytic interpretations, and the theory-based constructs considered during mixed methods integration.

Participants generally described GenAI as useful for obtaining explanations and supporting clinical learning, but not as sufficient on its own. They valued its apparent objectivity while recognizing that its training data might not reflect local patients or uncommon clinical presentations. Trust was conditional: students compared AI-generated output with supervisors’ views, their own clinical reasoning, or cases encountered in practice. When an output was incorrect, comparison with the clinical situation sometimes became a learning opportunity.

Explainability influenced whether students felt comfortable using the output. Reasoned explanations supported engagement, whereas answers without justification prompted hesitation. Privacy concerns focused on possible disclosure or reuse of patient information and, for some participants, the sense of being monitored. These concerns coexisted with continued GenAI use and shaped how cautiously students approached the technology.

Confidence developed through repeated use, but participants continued to place final judgment with the learner and clinical supervisor. Discussing AI-generated output with supervisors helped students compare the system’s reasoning with clinical expectations. Perceived control was therefore described as an active part of using GenAI rather than a simple decision to accept or reject its output.

**Table 2:** Qualitative themes derived from interviews with medical clerkship students, representative quotations, conceptual interpretations, and corresponding theory-based quantitative constructs.

Theme	Respondent quotes	Conceptual interpretation	Related variable
T1: Perceived fairness in AI decision-making	“AI seems fairer than my supervisor, but I worry the dataset doesn’t represent our local patients.”	Reflects concern about algorithmic objectivity and contextual fairness.	AI-perceived algorithmic fairness
T2: Algorithmic bias awareness	“Sometimes AI misses rare cases; I think it’s trained on typical Western data.”	Awareness of cultural and contextual limitations in AI datasets.	AI-perceived algorithmic fairness
T3: Trusting but verifying AI output	“I trust AI only after checking with my consultant’s opinion.”	Demonstrates calibrated trust and critical engagement.	AI-trust calibration literacy
T4: Learning from AI mistakes	“When AI gave a wrong result, I learned why by comparing with real cases.”	Reflective practice promotes deeper literacy in trust calibration.	AI-trust calibration literacy
T5: Understanding AI logic	“It helps when AI explains the reasoning behind its diagnosis.”	Perceived transparency enhances learning confidence.	AI-explainability quality exposure
T6: Struggling with nontransparent AI	“AI gives results without justification; I hesitate to rely on it.”	Lack of interpretability reduces engagement and self-efficacy.	AI-explainability quality exposure
T7: Privacy concerns about patient data	“I’m afraid our patients’ data could leak through the AI system.”	Highlights ethical awareness and sensitivity to data governance.	AI-privacy concern toward AI monitoring
T8: Feeling monitored by AI systems	“It feels like I’m constantly being observed by the system.”	AI surveillance may reduce autonomy and comfort.	AI-privacy concern toward AI monitoring
T9: Ethical reflection on data use	“We must ensure data isn’t reused without consent.”	Reinforces professional accountability in digital contexts.	AI-privacy concern toward AI monitoring
T10: Building confidence through practice	“At first, I was nervous, but after using AI daily, I got confident.”	Self-efficacy improves through repetitive, supportive use.	Self-efficacy in AI-generated usage
T11: AI as a learning partner	“AI helped me learn diagnostic reasoning faster.”	AI functions as a co-educator, enhancing learning autonomy.	Self-efficacy in AI-generated usage
T12: Balancing AI and human judgment	“AI assists with screening, but final judgment must be mine.”	Reflects perceived equilibrium between automation and human control.	Perceived AI self-control balance
T13: Human-AI collaboration in clinical tasks	“Discussing AI results with my supervisor made me understand both sides.”	Collaborative reasoning fosters confidence and ethical control.	Perceived AI self-control balance
T14: Patient safety awareness through AI	“AI reminders reduced small medication errors.”	Demonstrates how AI integration links to safer clinical practices.	Promotion of patient safety
T15: AI-driven infection monitoring	“AI flags unusual infection trends before we notice.”	Links AI insights to infection prevention strategies.	Ward-based infection-prevention practice
T16: Institutional support for safe AI use	“When the hospital trains us on AI ethics, we feel more secure.”	Organizational facilitation reinforces trust and responsible use.	Promotion of patient safety

Patient-safety themes appeared in accounts of medication-error reminders, recognition of unusual infection trends, and institutional training on safe AI use. These examples did not suggest that GenAI independently improved patient safety. Rather, participants understood its value as conditional on verification, supervision, and clear responsibility for clinical decisions. Across the themes, GenAI was experienced as a learning aid whose usefulness remained tied to explainability, critical review, privacy awareness, and continued human judgment.

### Phase 2: Quantitative Results

#### Participant Flow

Survey invitations were sent individually to 258 medical clerkship students through faculty representatives. Of these, 238 submitted the online questionnaire, corresponding to a response rate of 92.3% among the invited students. Twenty-four submissions were excluded because the screening responses did not confirm eligibility or the main questionnaire was incomplete. The remaining 214 responses were included in the PLS-SEM analysis, representing 89.9% (218/238) of the submitted questionnaires and 82.9% (214/258) of the students invited. The quantitative participant flow is shown in Multimedia Appendix 2, and the characteristics of the final analytic sample are presented in Table 1.

#### Recruitment

Recruitment and online survey data collection for the quantitative phase took place from February to March 2026. Because the survey was cross-sectional, no follow-up assessment was conducted.

#### Statistics and Data Analysis

##### Data Diagnostics

###### Missing Data

The SmartPLS execution log identified the input as a complete dataset. All 214 observations were used for each of the 32 indicators retained in the final measurement model. No item-level missing data were reported; therefore, missing completely at random (MCAR) testing, imputation, and other missing-data treatments were not required.

###### Distributions

The observed item values reported in the SmartPLS multivariate descriptives remained within the response range of their respective measures. Across the 32 retained indicators, skewness ranged from −0.938 to 0.434, and excess kurtosis ranged from −0.701 to 3.282. The Cramér-von Mises tests were statistically significant for all indicators (all *P*<.001), indicating departures from univariate normality. No normalizing transformation was applied. Model estimation was conducted using PLS-SEM, with statistical inference based on bootstrapping with 10,000 subsamples [56]. All 214 observations were retained in the analysis.

##### Data Summary

Descriptive statistics and correlations among the 8 construct scores are shown in Table 3. Complete construct scores were available for all 214 respondents. Because the measures used different response ranges, mean scores were interpreted within their respective scales and were not compared directly across constructs. The correlations are provided as descriptive information and do not replace the HTMT assessment reported in the “Measurement Model Estimates” section.

**Table 3:** Descriptive statistics and correlations among construct scores in the quantitative sample (n=214)^a^.

Constructs	Mean	SD	AI-PAF^b^	AI-TCL^c^	AI-EQE^d^	AI-PCAM^e^	SEAIGU^f^	PAISCB^g^	PPS^h^	NIFRDT^i^
AI-PAF	4.109	0.418	—	0.663	0.557	0.506	0.590	0.480	0.401	0.484
AI-TCL	3.994	0.447	0.663	—	0.684	0.693	0.677	0.454	0.410	0.616
AI-EQE	4.134	0.495	0.557	0.684	—	0.658	0.686	0.547	0.327	0.711
AI-PCAM	4.079	0.457	0.506	0.693	0.658	—	0.682	0.396	0.369	0.591
SEAIGU	4.186	0.449	0.590	0.677	0.686	0.682	—	0.364	0.429	0.606
PAISCB	3.662	0.621	0.480	0.454	0.547	0.396	0.364	—	0.077	0.569
PPS	4.127	0.647	0.401	0.410	0.327	0.369	0.429	0.077	—	0.196
NIFRDT	3.924	0.502	0.484	0.616	0.711	0.591	0.606	0.569	0.196	—

^a^AI-TCL was measured on a 7-point scale, PAISCB on a 6-point scale, and the remaining constructs on 5-point scales. Ward-based infection-prevention items (NIFRDT) were reverse-coded as specified in Multimedia Appendix 4; higher scores indicate better self-reported practice. Off-diagonal values are Pearson correlations among unstandardized partial least squares construct scores. The matrix is symmetric.

^b^AI-PAF: AI-perceived algorithmic fairness.

^c^AI-TCL: AI-trust calibration literacy.

^d^AI-EQE: AI-explainability quality exposure.

^e^AI-PCAM: AI-privacy concern toward AI monitoring.

^f^SEAIGU: self-efficacy in AI-generated usage.

^g^PAISCB: perceived AI self-control balance.

^h^PPS: promotion of patient safety.

^i^NIFRDT is the construct code used for ward-based infection-prevention practice.

##### Model Fit

For the standardized solution, the saturated model had a standardized root mean square residual (SRMR) of 0.082 and a normed fit index (NFI) of 0.702, while the estimated model had an SRMR of 0.089 and an NFI of 0.691. The corresponding d_ULS values were 3.539 and 4.223, and the d_G values were 1.283 and 1.348, respectively. The observed SRMR, d_ULS, and d_G values were above their 99% bootstrap quantiles for both models. Thus, the bootstrap results did not support exact model fit.

Pairwise correlation residuals were not used as a separate criterion for model retention. Consistent with the predictive orientation of PLS-SEM, the model was evaluated primarily through outer loadings, construct reliability and validity, collinearity, explained variance, structural path estimates, and out-of-sample predictive performance [56]. Indicator- and construct-level measurement results, including SEAIGU5, which assessed students’ perceived ability to keep up with changes in GenAI service features, and the borderline HTMT CIs for 3 construct pairs, are reported in the “Measurement Model Estimates” section. All findings reported below are based on the final 32-indicator model described in the “Respecification” section.

##### Respecification

The initial measurement model contained 35 indicators, including 6 indicators for promotion of patient safety. During the initial assessment, PPS4, PPS5, and PPS6 had low outer loadings. These indicators were removed after their contribution to indicator reliability, construct reliability, convergent validity, and conceptual coverage had been considered [56]. The removal did not materially narrow the construct because promotion of patient safety remained represented by PPS1-PPS3. In the reestimated model, this construct had a Cronbach α of 0.841, composite reliability of 0.899, and AVE of 0.748.

The final measurement model contained 32 indicators. SEAIGU5 had an outer loading of 0.543 but was retained because its loading remained above 0.40; the self-efficacy construct continued to meet the reliability and convergent-validity criteria, and the item captured students’ perceived ability to keep up with changes in GenAI features [56].

No construct, hypothesis, or structural path was added, removed, or redirected during respecification. The change was confined to the measurement model and was completed before hypothesis testing, predictive assessment, moderation analysis, and importance-performance map analysis. All subsequent analyses used the final 32-indicator model. The retained indicators and final measurement-model estimates are reported in Multimedia Appendix 6.

##### Estimates

###### Measurement Model Estimates

The final measurement model was evaluated using standardized outer loadings, Cronbach α, rho_A, composite reliability, AVE, and the HTMT [56]. All retained indicators except SEAIGU5 had outer loadings above 0.70, ranging from 0.711 to 0.913. SEAIGU5 had an outer loading of 0.543 and was retained for the reasons described in the “Respecification” section.

All 8 constructs met the stated criteria for internal consistency and convergent validity. Cronbach α values ranged from 0.747 to 0.887, composite reliability values ranged from 0.840 to 0.926, and AVE ranged from 0.567 to 0.806. For promotion of patient safety, rho_A was 0.972 and exceeded its composite reliability of 0.899. Because this construct contained only 3 retained indicators, its internal consistency was interpreted mainly from Cronbach α (0.841) and composite reliability (0.899), together with an AVE of 0.748. The indicator loadings and construct-level estimates are reported in Multimedia Appendices 4 and 6.

Discriminant validity was examined using HTMT point estimates and bootstrapped 95% CIs. All point estimates were below 0.90, and none of the CIs included 1.00. The highest estimate was found between AI-trust calibration literacy and AI-perceived algorithmic fairness (HTMT=0.861, 95% CI 0.765-0.940). The upper confidence limit also exceeded 0.90 for AI-trust calibration literacy and AI-privacy concern toward AI monitoring (HTMT=0.830, 95% CI 0.736-0.909) and for ward-based infection-prevention practice and AI-explainability quality exposure (HTMT=0.845, 95% CI 0.770-0.909). These 3 pairs were therefore interpreted cautiously rather than treated as unequivocally distinct. The complete HTMT results are presented in Multimedia Appendix 5.

###### Structural Model Estimates

The inner variance inflation factor values ranged from 1.246 to 2.786, indicating that collinearity was not a concern in the structural model. The 4 GenAI-related antecedents explained 61.6% of the variance in self-efficacy in AI-generated usage (*R*²=0.616, adjusted *R*²=0.609) and 34.1% of the variance in PAISCB (*R*²=0.341, adjusted *R*²=0.328). The complete model explained 51.9% of the variance in ward-based infection-prevention practice (*R*²=0.519, adjusted *R*²=0.507).

Statistical inference was based on 10,000 bootstrap subsamples, one-tailed tests at α=.05, and 90% BC CIs. Table 4 presents the unstandardized and standardized path estimates, bootstrap SEs and CIs, exact *P* values, and decisions for the individual paths. The findings varied across the 3 hypothesis groups. Among the appraisal-mechanism paths specified in H1-H8, 5 met the prespecified directional and statistical criteria, while H7 was statistically significant in the opposite direction. Both mechanism-outcome paths specified in H9 and H10 met the prespecified criteria. Among the 2 interaction paths, H11 met the nominal criterion, whereas H12 did not.

Self-efficacy in AI-generated usage had the largest association with ward-based infection-prevention practice (β=0.477, 90% BC CI 0.355-0.604; *f*²=0.327; *P*<.001), followed by PAISCB (β=0.407, 90% BC CI 0.270-0.518; *f*²=0.264; *P*<.001).

**Table 4:** Structural model hypothesis testing results^a^.

Hypothesis	Structural path	b (SE)	90% BC^b^ CI for b	β (SE)	90% BC CI for β	*P* value	Decision
H1	AI-PAF^c^ → SEAIGU^d^	0.188 (0.076)	0.062 to 0.312	0.185 (0.070)	0.062 to 0.294	.004	Supported
H2	AI-PAF → PAISCB^e^	0.364 (0.151)	0.137 to 0.625	0.236 (0.098)	0.066 to 0.391	.008	Supported
H3	AI-TCL^f^ → SEAIGU	0.165 (0.092)	0.022 to 0.323	0.165 (0.085)	0.021 to 0.298	.03	Supported
H4	AI-TCL → PAISCB	0.032 (0.166)	−0.221 to 0.313	0.033 (0.119)	−0.169 to 0.223	.39	Not supported
H5	AI-EQE^g^ → SEAIGU	0.256 (0.083)	0.124 to 0.402	0.279 (0.084)	0.136 to 0.412	<.001	Supported
H6	AI-EQE → PAISCB	0.500 (0.124)	0.305 to 0.704	0.401 (0.096)	0.242 to 0.559	<.001	Supported
H7	AI-PCAM^h^ → SEAIGU	0.289 (0.083)	0.148 to 0.419	0.294 (0.081)	0.162 to 0.429	<.001	Not supported^i^
H8	AI-PCAM → PAISCB	−0.008 (0.155)	−0.305 to 0.212	−0.013 (0.108)	−0.187 to 0.168	.45	Not supported
H9	SEAIGU → NIFRDT^j^	0.543 (0.073)	0.426 to 0.663	0.477 (0.075)	0.355 to 0.604	<.001	Supported
H10	PAISCB → NIFRDT	0.329 (0.065)	0.213 to 0.422	0.407 (0.077)	0.270 to 0.518	<.001	Supported
H11	SEAIGU × PPS^k^ → NIFRDT	0.214 (0.104)	0.058 to 0.376	0.122 (0.071)	0.011 to 0.237	.04	Supported
H12	PAISCB × PPS → NIFRDT	−0.118 (0.096)	−0.261 to 0.035	−0.097 (0.085)	−0.216 to 0.056	.13	Not supported

^a^SEs and 90% bias-corrected CIs were derived from 10,000 bootstrap subsamples. H9 and H10 estimates are conditional direct effects evaluated at the mean of promotion of patient safety. *P* values and decisions were based on standardized, 1-tailed bootstrap tests at α=.05. The *P* values are path-specific and unadjusted; no formal multiplicity adjustment was applied. Decisions concern individual directional hypotheses, not an omnibus test of the model, and findings near the nominal threshold should be interpreted cautiously. b is the unstandardized coefficient and β is the standardized coefficient.

^b^BC: bias-corrected.

^c^AI-PAF: AI-perceived algorithmic fairness.

^d^SEAIGU: self-efficacy in AI-generated usage.

^e^PAISCB: perceived AI self-control balance.

^f^AI-TCL: AI-trust calibration literacy.

^g^AI-EQE: AI-explainability quality exposure.

^h^AI-PCAM: AI-privacy concern toward AI monitoring.

^i^H7 was classified as not supported because the observed association was positive rather than negative as hypothesized.

^j^NIFRDT is the construct code used for ward-based infection-prevention practice.

^k^PPS: promotion of patient safety.

###### Specific Indirect Effects

The structural model did not include direct paths from the 4 GenAI-related antecedents to ward-based infection-prevention practice. The results are therefore reported as specific conditional indirect effects through self-efficacy in AI-generated usage and PAISCB, without assigning a mediation type. Because promotion of patient safety moderated both mediator-outcome paths, the indirect effects were evaluated at the mean level of the moderator. Inference was based on 10,000 bootstrap subsamples, 1-tailed tests at α=.05, and 90% BC CIs. Bootstrapping was used because an indirect effect is a product of path coefficients and its sampling distribution need not be normal. The *P* values reported below are from the standardized bootstrap estimates.

At the mean level of promotion of patient safety, all 4 conditional indirect effects through self-efficacy in AI-generated usage were positive and statistically significant: AI-explainability quality exposure (b=0.139, SE=0.053, 90% BC CI 0.069-0.242; β=0.133, SE=0.052, 90% BC CI 0.058-0.228; *P*=.006), AI-perceived algorithmic fairness (b=0.102, SE=0.045, 90% BC CI 0.034-0.182; β=0.088, SE=0.038, 90% BC CI 0.030-0.155; *P*=.01), AI-privacy concern toward AI monitoring (b=0.157, SE=0.046, 90% BC CI 0.088-0.242; β=0.140, SE=0.042, 90% BC CI 0.075-0.214; *P*<.001), and AI-trust calibration literacy (b=0.090, SE=0.053, 90% BC CI 0.016-0.186; β=0.079, SE=0.042, 90% BC CI 0.012-0.147; *P*=.03).

The conditional indirect effects through PAISCB were positive and statistically significant for AI-explainability quality exposure (b=0.164, SE=0.059, 90% BC CI 0.085-0.277; β=0.163, SE=0.057, 90% BC CI 0.084-0.275; *P*=.002) and AI-perceived algorithmic fairness (b=0.120, SE=0.052, 90% BC CI 0.049-0.228; β=0.096, SE=0.042, 90% BC CI 0.036-0.177; *P*=.01). The corresponding effects were not statistically significant for AI-trust calibration literacy (b=0.011, SE=0.054, 90% BC CI −0.070 to 0.097; β=0.014, SE=0.047, 90% BC CI −0.067 to 0.088; *P*=.39) or AI-privacy concern toward AI monitoring (b=−0.003, SE=0.050, 90% BC CI −0.100 to 0.070; β=−0.005, SE=0.043, 90% BC CI −0.076 to 0.065; *P*=.45).

###### Interaction Effects

Promotion of patient safety was examined as a moderator of the associations between the 2 human-centered constructs and ward-based infection-prevention practice. Its constituent main effect was retained in the outcome equation but was not statistically significant (β=−0.049, 90% BC CI −0.149 to 0.041; *P*=.20).

The interaction between self-efficacy in AI-generated usage and promotion of patient safety was small and positive (β=0.122, 90% BC CI 0.011-0.237; *f*²=0.028; *P*=.04), meeting the prespecified nominal criterion for H11. Given its proximity to the significance threshold and small effect size, this result was interpreted cautiously. The conditional association between self-efficacy and ward-based infection-prevention practice increased from β=0.355 at a low level of promotion of patient safety (−1 SD; 90% BC CI 0.167-0.562), to β=0.477 at its mean (90% BC CI 0.355-0.604), and β=0.599 at a high level (+1 SD; 90% BC CI 0.460-0.723). The simple slopes therefore followed the direction of the positive interaction.

The interaction between PAISCB and promotion of patient safety was negative but not statistically significant (β=−0.097, 90% BC CI −0.216 to 0.056; *f*²=0.018; *P*=.13), and H12 was not supported. The corresponding conditional coefficients were β=0.504 at −1 SD (90% BC CI 0.229-0.675), β=0.407 at the mean (90% BC CI 0.270-0.518), and β=0.311 at +1 SD (90% BC CI 0.185-0.443). Although the coefficients became smaller as promotion of patient safety increased, this pattern should not be interpreted as evidence of moderation because the interaction term was not statistically significant. The interaction estimates are included in Table 4, and both simple-slope patterns are shown in Figure 1.

**Figure 1:**
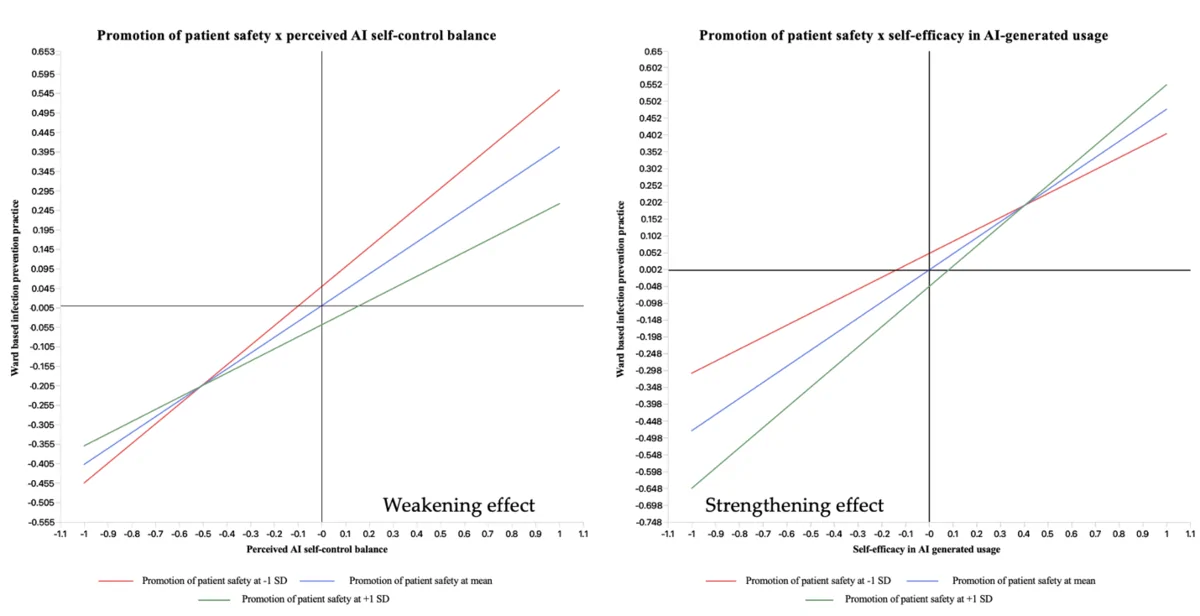
Simple slope plots showing the moderating role of promotion of patient safety in the associations between self-efficacy in AI-generated usage, perceived AI self-control balance, and ward-based infection-prevention practice.

##### Predictive Assessment

All 3 endogenous constructs had positive Q²predict values. The value was 0.588 for self-efficacy in AI-generated usage, 0.298 for PAISCB, and 0.486 for ward-based infection-prevention practice. These results indicated that the PLS-SEM predictions performed better than the corresponding naïve prediction benchmark for each construct [56].

Predictive performance was further examined using the CVPAT. Compared with the indicator average benchmark, the PLS-SEM model produced lower loss values for ward-based infection-prevention practice (average loss difference=−0.123, *P*<.001), PAISCB (average loss difference=−0.117, *P*=.001), and self-efficacy in AI-generated usage (average loss difference=−0.118, *P*<.001). The overall average loss difference was −0.119 (*P*<.001), indicating better predictive performance than the indicator average benchmark.

The results were less consistent when the PLS-SEM model was compared with the linear model benchmark. PLS-SEM produced lower loss values for ward-based infection-prevention practice (average loss difference=−0.038, *P*=.02) and self-efficacy in AI-generated usage (average loss difference=−0.021, *P*=.02). For PAISCB, however, the PLS-SEM loss was higher than the linear model loss (average loss difference=0.075, *P*=.004). At the overall-model level, the loss difference was 0.009 (*P*=.37), indicating no statistically significant difference between PLS-SEM and the linear model benchmark.

Taken together, the model showed predictive usefulness relative to the indicator average benchmark but did not consistently outperform the linear model benchmark. The construct-specific result for PAISCB was therefore considered when interpreting findings involving this construct [56,57,60]. The complete CVPAT results are presented in Table 5.

**Table 5:** Construct-specific and overall predictive performance of the partial least squares structural equation modeling (PLS-SEM) model compared with indicator average (IA) and linear model (LM) benchmarks using the Cross-Validated Predictive Ability Test (CVPAT).

Variable	PLS-SEM vs IA					PLS-SEM vs LM				
	PLS loss	IA loss	Average loss difference	*P* value	PLS loss		LM loss	Average loss difference	*P* value	
Ward-based infection-prevention practice	0.365	0.488	–0.123	<.001	0.365		0.403	–0.038	.02	
Perceived AI self-control balance	0.542	0.658	–0.117	.001	0.542		0.466	0.075	.004	
Self-efficacy in AI-generated usage	0.202	0.320	–0.118	<.001	0.202		0.223	–0.021	.02	
Overall	0.370	0.489	–0.119	<.001	0.370		0.361	0.009	.37	

##### Importance-Performance Map Analysis

Importance-performance map analysis was conducted to identify indicators that were both relevant to ward-based infection-prevention practice and actionable for clerkship education. Following Ringle and Sarstedt [58], importance was interpreted from the total effects in the PLS-SEM model, while performance was interpreted from rescaled indicator mean scores. The response scale length for each construct is reported in Multimedia Appendix 3 to support transparency in performance rescaling. The IPMA results are shown in Figure 2.

**Figure 2:**
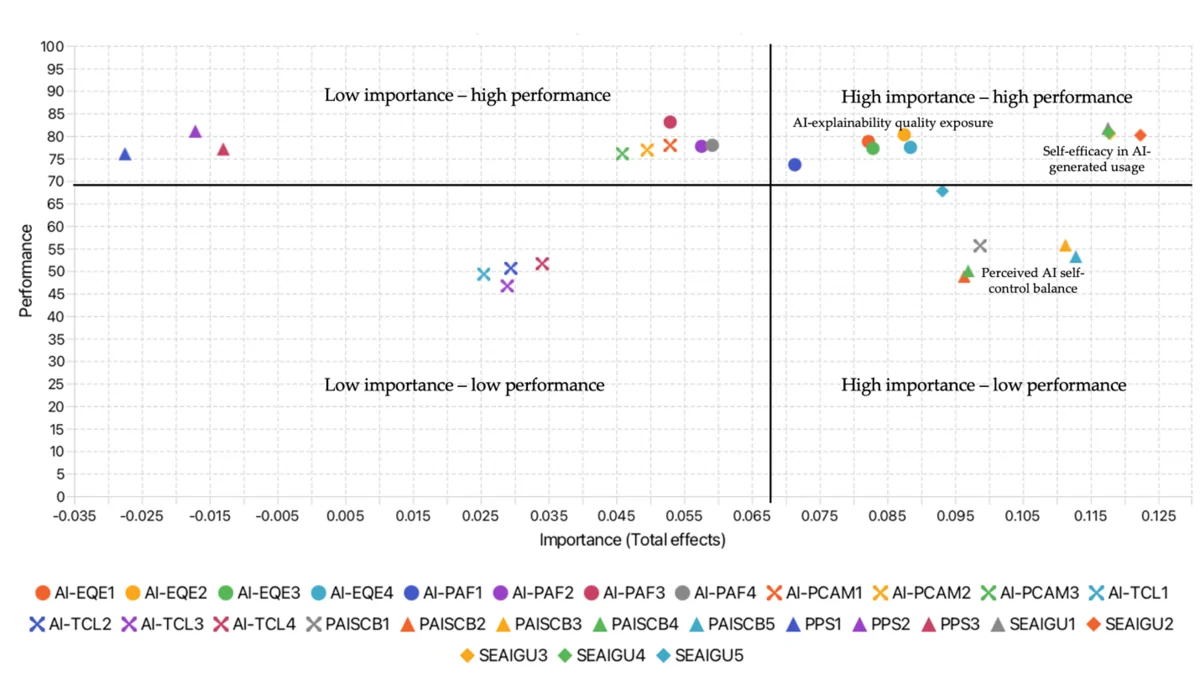
Importance-performance map analysis of indicators associated with ward-based infection-prevention practice among medical clerkship students from 5 private universities in the Jakarta Metropolitan Area, Indonesia. AI-EQE: AI-explainability quality exposure; AI-PAF: AI-perceived algorithmic fairness; AI-PCAM: AI-privacy concern toward AI monitoring; AI-TCL: AI-trust calibration literacy; PAISCB: perceived AI self-control balance; PPS: promotion of patient safety; SEAIGU: self-efficacy in AI-generated usage.

The indicator-level IPMA results are shown in Figure 2. All 4 indicators of AI-explainability quality exposure were located in the high-importance and high-performance area. Four of the 5 self-efficacy indicators (SEAIGU1-SEAIGU4) showed the same pattern, whereas SEAIGU5 had high importance but performance slightly below the reference value. These findings suggest that explainability quality exposure and most aspects of self-efficacy were relative strengths among the respondents.

All 5 indicators of PAISCB were located in the high-importance and lower-performance area, making this construct the most consistent priority area in the map. The AI-trust calibration literacy indicators showed comparatively low importance and low performance. The AI-privacy concern indicators and 3 of the 4 AI-perceived algorithmic fairness indicators had lower importance but relatively high performance, while AI-PAF1 was located in the high-importance and high-performance area.

The promotion of patient safety indicators showed high performance but low or negative importance. Because promotion of patient safety was specified as a moderator rather than a direct predictor, these indicator positions were not interpreted as direct targets for educational intervention. The priority identified for PAISCB should also be interpreted cautiously because the construct-specific CVPAT showed that the PLS-SEM model did not outperform the linear benchmark for this construct.

#### Integration of Qualitative and Quantitative Findings

##### Convergence, Expansion, and Divergence

At the second stage of integration, following the quantitative analysis, the qualitative themes in Table 2 were compared with the structural model results in Table 4. This comparison examined whether the interview accounts converged with, added context to, or differed from the quantitative findings.

The clearest convergence concerned explainability. Participants were more confident using GenAI when its responses included understandable reasoning and were more hesitant when answers were presented without justification. In the quantitative phase, explainability quality exposure was positively associated with self-efficacy in AI-generated usage (β=0.279, *P*<.001) and PAISCB (β=0.401, *P*<.001). Fairness showed a similar pattern. Participants valued the apparent objectivity of GenAI but also questioned whether its outputs adequately represented local patients and clinical settings. Perceived algorithmic fairness was positively associated with both self-efficacy (β=0.185, *P*=.004) and PAISCB (β=0.236, *P*=.008).

The qualitative findings added context to the more selective result for trust calibration literacy. Participants described checking AI-generated output against supervisors’ opinions, clinical reasoning, and actual patient cases. Some also reported learning from incorrect AI responses. In the quantitative phase, trust calibration literacy was associated with self-efficacy (β=0.165, *P*=.03), but not with PAISCB (β=0.033, *P*=.39). The ability to question and verify AI output therefore corresponded with greater confidence in using GenAI, but not necessarily with a stronger perception of control over the interaction.

Privacy concern showed the clearest divergence from the hypothesized direction. Participants expressed concern about patient-data leakage, unauthorized data reuse, and monitoring, but some continued to use GenAI because it remained useful for clinical and academic learning. Privacy concern was positively associated with self-efficacy (β=0.294, *P*<.001), contrary to the negative association proposed in H7, and was not associated with PAISCB (β=−0.013, *P*=.45). Thus, the two phases were not contradictory. Both showed that privacy concern could coexist with continued and confident GenAI use; the divergence concerned the direction proposed in H7.

The findings concerning human agency also showed convergence with some qualification. Participants described gaining confidence through repeated use while maintaining that final judgment should remain with the learner and supervisor. Quantitatively, self-efficacy (β=0.477, *P*<.001) and PAISCB (β=0.407, *P*<.001) were positively associated with self-reported ward-based infection-prevention practice. The specific indirect effects further showed that self-efficacy carried the associations of all 4 GenAI-related antecedents, whereas PAISCB carried only the associations of explainability and fairness. A small positive interaction was observed between promotion of patient safety and self-efficacy, whereas the corresponding interaction involving PAISCB was not observed, as shown in Figure 1. The interview findings provided context for the importance of safety orientation and supervision, but were not treated as independent evidence of statistical moderation.

## Discussion

### Principal Findings

This study examined how medical clerkship students used and interpreted GenAI in a safety-critical learning environment. The qualitative phase identified 4 GenAI-related domains that shaped students’ learning experiences: perceived algorithmic fairness, trust calibration literacy, explainability quality exposure, and privacy concern toward AI monitoring. These domains reflected how students appraised GenAI when using it alongside clinical learning, documentation, and patient safety–related tasks during ward rotations. In the quantitative phase, these domains were examined as antecedents of 2 human-centered mechanisms, namely self-efficacy in AI-generated usage and PAISCB, and their associations with self-reported ward-based infection-prevention practice. Self-efficacy carried significant indirect effects from all 4 antecedents, whereas PAISCB carried only those of explainability and fairness. This asymmetry indicates that self-efficacy was the more consistent pathway linking students’ appraisals of GenAI to self-reported infection-prevention practice. The two phases follow a single social cognitive logic, in which students’ appraisals of GenAI relate to safety-oriented practice mainly through their beliefs about their own capabilities rather than through acceptance alone [24]. GenAI-supported patient-safety learning in clerkship depends on access to AI tools and on how students understand, verify, and regulate AI-generated output within supervised workplace-based learning [2,4,7,8,39].

Among the GenAI-related antecedents, explainability quality exposure showed the most consistent association with both self-efficacy in AI-generated usage and PAISCB. Perceived algorithmic fairness was also positively associated with both mediators. The qualitative interviews showed the same pattern. Several participants described GenAI as useful when they could understand how an answer was produced, especially when preparing subjective-objective-assessment-plan (SOAP) notes, revising documentation, or organizing oral case presentations during ward learning. In this context, explainability functioned as a practical learning support that helped students compare AI-generated output with clinical expectations instead of accepting it as a final answer [30-32]. Fairness also appeared to support engagement with GenAI. When AI outputs were perceived as reasonable, consistent, and procedurally acceptable, students were more willing to use them during learning tasks related to clinical reasoning and patient safety. However, perceived fairness should not be equated with actual freedom from bias, particularly because AI-generated outputs may remain sensitive to training data, task context, and automation-related overconfidence [22,23,25,26].

The findings for trust calibration literacy were more selective. Trust calibration literacy was associated with self-efficacy in AI-generated usage but was not meaningfully associated with PAISCB. This distinction indicates that knowing when to question AI may strengthen students’ confidence in using GenAI, but does not automatically create a stronger sense of control over the interaction. In clerkship settings, students may understand the need to verify AI output while still having limited time, feedback, or structured opportunities to practice verification during ward routines. Research on automation bias and cognitive forcing supports this interpretation. Reflective engagement requires task design, supervision, and feedback that help learners decide whether AI output should be accepted, revised, or rejected [22,23,27-29,61].

The privacy concern finding also requires careful interpretation. Privacy concern toward AI monitoring was positively associated with self-efficacy in AI-generated usage, although the hypothesized direction was negative. The finding was therefore directionally reversed rather than statistically null. One possible explanation is risk-aware engagement. Students with greater privacy concerns may have paid closer attention to the information they entered, how prompts were framed, and when GenAI use was inappropriate. The interviews provide some context for this interpretation. Several students described feeling monitored by AI systems. They expressed concern about the responsible use of patient-related data (Table 2), yet these concerns did not lead them to discontinue GenAI use. Instead, they described using it more carefully. Privacy paradox research similarly suggests that people may continue to use digital tools despite privacy concerns when the perceived benefits are immediate and practical [33,34,62,63]. Other explanations cannot be ruled out. Because privacy concern and self-efficacy were measured in the same self-report survey, the observed association may partly reflect shared method variance or a general tendency to endorse questionnaire items. The AI-PCAM items may also have reflected privacy awareness among students who were already more confident in using GenAI, rather than concern that discouraged its use. The cross-sectional design cannot distinguish among these possibilities. For clerkship education, privacy concern may become educationally useful when it is connected to data governance, professional accountability, and supervised guidance on safe AI use [4,8,9].

The findings involving PAISCB should be interpreted within the scope of the construct and its measurement. PAISCB was positively associated with self-reported ward-based infection-prevention practice. However, the construct-specific CVPAT showed that the PLS-SEM model did not outperform the linear benchmark for this construct [56,57,60]. The items captured students’ perceived control over GenAI interaction [38]. This construct was associated with self-reported infection-prevention practice but did not measure clinical oversight or accountability; safe use still depends on supervision, protocol alignment, and clear boundaries of responsibility [4,8,22,23,39].

Across phases, the findings converged for explainability and fairness, expanded the interpretation of trust calibration, and helped explain the positive association between privacy concern and self-efficacy. Because the qualitative findings informed development of the quantitative framework, these correspondences were interpreted as cross-phase coherence rather than independent confirmation [45]. Together, the findings position GenAI as a supervised educational practice linked to patient-safety learning rather than technology acceptance alone [2,4,6,9,64].

### Theoretical Implications

This study contributes to the theoretical understanding of GenAI-supported learning in medical clerkship by positioning self-efficacy and PAISCB as human-centered mechanisms, rather than as general indicators of technology acceptance. The findings are organized around a single social cognitive frame, in which students’ appraisals of AI-generated output relate to learning behavior mainly through their beliefs about their own capabilities rather than through acceptance alone [24]. Within this frame, self-efficacy reflects students’ belief in their capacity to organize and perform learning tasks with AI support [24], and it emerged as the mechanism most clearly linked to self-reported infection-prevention practice [65,66].

The reasoning behind this link can be stated more precisely. Infection-prevention practice on the ward is not a single decision but a set of repeated, effortful routines carried out under supervision and time pressure, including hand hygiene, use of protective equipment, and documentation [3,39]. Social cognitive theory holds that self-efficacy influences whether learners initiate such effortful actions, how much effort they sustain, and how they persist when a task is demanding [24]. A student may use AI to clarify a precaution, check a protocol, or prepare a case. When the output is verified against clinical findings and supervisor guidance, that support is more likely to inform consistent practice. Avoiding AI or relying on it without checking offers less support for such practice. In theoretical terms, the contribution of self-efficacy operates less through the technology itself than through the learner’s willingness to translate AI support into deliberate, verified action within supervised work. This extends self-efficacy theory into a clinical education context in which learners use AI assistance while still working under supervision, clinical uncertainty, and patient-safety expectations.

Within the same agentic view, the findings refine the theoretical role of autonomy in GenAI-supported learning. Self-determination theory serves here as a complementary lens, emphasizing autonomy and competence as psychological conditions for learning [67]. PAISCB can be viewed as a learning-related form of agency in human-AI interaction [38]. It does not imply independent control over patient-facing decisions, which remain dependent on supervision, verification, and institutional safeguards.

These individual-level mechanisms operate within a wider educational context, which workplace-based learning and competency-based medical education help to specify. Workplace-based learning views clinical education as participation in real clinical work, where supervision, feedback, and graded responsibility shape how students develop professional competence [39,68,69]. Competency-based medical education similarly emphasizes progression toward defined competencies and readiness for safe practice rather than learning based only on time spent in training [70]. By examining self-reported ward-based infection-prevention practice as a safety-oriented learning outcome, this study links GenAI-related perceptions to the practical routines of clerkship learning. GenAI is positioned as a tool whose educational value depends on how it is embedded within ward supervision, feedback, protocol use, and patient-safety routines.

Complementing this individual-level account, the findings also support a situated view of GenAI in medical education. From this perspective, learning is shaped by individual cognition and by interactions among learners, supervisors, digital tools, institutional protocols, and clinical tasks [71]. Explainability and perceived fairness are not solely technical attributes of AI systems. In clerkship learning, they also function as sociocognitive resources that influence whether students are willing to bring AI-generated output into documentation, case preparation, and infection-prevention reasoning. This framing helps explain why the same AI tool may support learning in one setting but encourage superficial use in another, depending on how the tool is situated within supervision and local clinical practice.

The pattern of supported and unsupported paths provides a boundary condition for theories of human-AI learning. Trust calibration literacy was associated with self-efficacy but not with PAISCB, and general promotion of patient safety did not strengthen the association between PAISCB and ward-based infection-prevention practice. The results suggest that awareness of AI limitations and broad safety messages may be insufficient unless they are translated into supervised verification practices. Research on automation bias, cognitive forcing, and the cognitive atrophy paradox supports this interpretation. Intelligent systems may improve efficiency while reducing active cognitive engagement when users offload reasoning to AI [22,23,61,72]. In clerkship education, accounts of GenAI-supported learning should include the educational conditions that preserve students’ reasoning, verification, and professional responsibility.

Overall, GenAI is best understood as part of a supervised learning ecology whose educational value depends on verification, feedback, and institutional safeguards.

### Educational and Patient-Safety Implications

The IPMA placed PAISCB, rather than self-efficacy as a whole, as the main area with room for improvement. All PAISCB indicators combined high importance with lower performance, whereas 4 of the 5 self-efficacy indicators and all AI-explainability quality exposure indicators showed high importance and high performance. The more immediate educational need may therefore be to help students retain control over their interaction with GenAI—for example, by setting clear goals, deciding how prompts are used, and checking AI-generated outputs—rather than focusing only on confidence in using the technology. This interpretation remains cautious because the PLS-SEM model did not outperform the linear benchmark for perceived AI self-control balance in the construct-specific CVPAT [56,58,60].

GenAI should be introduced as a provisional learning support, not as a substitute for clinical judgment. Training in verification, patient-data protection, and appropriate GenAI use should begin early in medical education and be reinforced during clerkship. Students can use GenAI to organize infection prevention and control guidance, summarize isolation criteria, clarify the rationale behind precautions, or prepare an initial interpretation before ward discussion. The educational value of these activities depends on verification against patient findings, institutional protocols, and supervisor feedback [70,73]. Case discussions provide a practical setting for this process. Supervisors should ask how students used GenAI. They should also ask which outputs were accepted or rejected and how the final interpretation was checked against clinical and infection-prevention standards [70]. Educational activities can translate this form of control into observable practices, including prompt formulation, source checking, comparison with institutional protocols, supervisor confirmation, and reflection on uncertainty [70,73].

The comparatively strong performance of explainability quality exposure indicates that many students already value AI outputs that are understandable and usable for learning. Explainability is insufficient when it remains disconnected from supervised clinical behavior [30,58]. Medical schools and teaching hospitals should develop clerkship guidance for GenAI use, including patient-data protection rules, examples of acceptable and unacceptable prompts, structured verification steps, and supervisor-led reflection on AI-generated outputs. This guidance would embed GenAI-supported learning in patient-safety education and connect infection-prevention learning with clinical accountability and institutional standards [73-76].

### Limitations and Future Research

This study should be interpreted in light of several limitations. First, the quantitative phase used a cross-sectional design; therefore, the observed associations cannot be interpreted as causal relationships. The sequential mixed methods design allowed the findings from both phases to be considered together; however, the survey data captured students’ perceptions and self-reported practices at one point in time. The reported model was retained as the primary model because its structure was developed through the sequential integration of the qualitative findings with social cognitive theory and prior evidence and was finalized before quantitative data collection and analysis. It was therefore not selected after comparing alternative arrangements against the observed quantitative results. Alternative arrangements of the constructs were not estimated. The findings support the reported associations within the specified model but do not establish that it was the only plausible representation of the observed relationships. In addition, the structural hypotheses were evaluated as directional paths using *P* values and CIs, without a formal adjustment for the number of paths tested; results close to the significance threshold warrant particular caution. Future studies should use longitudinal or interventional designs to examine whether structured GenAI training, supervised verification exercises, or infection-prevention learning modules produce sustained changes in students’ AI-related self-efficacy and ward-based patient-safety learning.

Second, the study relied on self-reported ward-based infection-prevention practice rather than direct observation, supervisor ratings, or clinical infection indicators. This approach was appropriate for examining students’ perceived learning and reported practice during clerkship, but it remains subject to recall bias and social desirability bias. Procedural steps were taken to reduce common method bias, including anonymity, screening procedures, and clear response instructions; however, residual common method bias cannot be fully excluded in self-report survey research [55]. Future research should combine student-reported outcomes with observed behaviors, supervisor assessments, simulation-based performance, or audit-based indicators of infection-prevention practice.

Third, the study was conducted among medical clerkship students from 5 private universities in the Jakarta Metropolitan Area using purposive sampling. This sampling strategy ensured that respondents had direct experience using GenAI in clinical learning, but it also limits the generalizability of the findings to other medical schools, public universities, rural training sites, or clerkship systems with different supervision models and institutional policies. The qualitative phase also included 8 informants, which was appropriate for contextual exploration based on information power and the contextual range and depth of the interviews, but was not intended to represent the full diversity of medical clerkship experiences. Future studies should include larger and more diverse samples across public and private institutions, multiple regions, and different types of teaching hospitals. In addition, because participation required prior frequent or very frequent GenAI use, self-selection bias may have occurred, as students who were more digitally engaged or more interested in GenAI-supported learning may have been more likely to participate. Accordingly, the quantitative estimates should be read as conditional on this group of frequent GenAI users rather than as estimates for the broader clerkship population. Restricting the sample in this way may also affect the magnitude of the observed associations, although the direction of this effect, whether attenuation or strengthening, cannot be determined from the present data. Although 238 of the 258 individually invited students submitted the survey, the available data did not permit a comparison between respondents and nonrespondents; some nonresponse bias therefore remains possible.

Fourth, perceived AI self-control balance did not measure supervised verification, human accountability, protocol alignment, or clinical responsibility [38]. Its construct-specific CVPAT also showed less consistent predictive performance than that of self-efficacy [56,57,60]. Future studies should refine this construct by developing or adapting items that more directly capture supervised verification, human accountability, protocol alignment, and clinical responsibility in AI-supported patient-safety learning.

Fifth, the mixed methods integration has limitations that qualify the interpretation of the correspondences between the two phases. Although the themes were developed inductively, the interview guide was informed by the study objectives, theory, and prior literature, and the subsequent abductive building stage considered the themes alongside established theoretical constructs. This process may have made the analysis more attentive to patterns that corresponded with the selected theories and measures. A formal discrepant-case analysis was not conducted, although contrasting participant accounts and quantitative paths that were unsupported or opposite to the hypothesized direction were retained in the interpretation. Because the qualitative findings contributed to the development of the quantitative framework, agreement across phases should be understood as theory-informed coherence rather than independent confirmation. Future studies could strengthen mixed methods legitimacy through explicit joint displays, formal discrepant-case analysis, and further examination of explanations that do not fit the initial theoretical framework [45,49].

Sixth, although none of the bootstrapped HTMT CIs included 1.00, the upper limits exceeded 0.90 for 3 construct pairs. This finding indicates some empirical overlap in the present sample, particularly between AI-trust calibration literacy and AI-perceived algorithmic fairness. These constructs were retained because they address different aspects of students’ appraisal of GenAI, but findings involving the borderline pairs should be interpreted cautiously. Further studies should reassess their empirical separation in more diverse clerkship populations. AI-trust calibration literacy concerns how students evaluate and calibrate their reliance on AI-generated conclusions, whereas AI-perceived algorithmic fairness concerns judgments of procedural fairness and nondiscrimination in AI-based services.

Finally, heterogeneity in clerkship environments may have influenced the observed relationships. Students rotated through different hospitals, clinical departments, patient case mixes, supervision styles, and infection-prevention policy environments. This variation reflects the real-world nature of workplace-based medical education, but it also introduces contextual differences that were not fully accounted for in the present model. Because university identifiers were not retained in the respondent-level dataset, clustering by institution and differences among the 5 participating universities could not be examined. Future research should retain nonidentifying institutional codes and examine whether institutional support, supervisor readiness, clinical workload, and department-level safety culture influence the educational value of GenAI during clerkship. Such studies may help clarify how GenAI can be integrated into patient-safety learning without weakening students’ clinical reasoning, verification habits, or professional responsibility.

### Conclusion

In the interviews, students valued GenAI when its responses were understandable and could be checked against clinical knowledge and supervisor guidance, while also raising concerns about bias, privacy, and the need to retain human judgment. In the quantitative phase, explainability quality exposure and perceived algorithmic fairness were associated with students’ self-efficacy and perceived control in using GenAI. Self-efficacy was the most consistent pathway linking all 4 antecedents to self-reported ward-based infection-prevention practice, whereas perceived AI self-control balance carried only the effects of explainability and fairness. The positive association between privacy concern and self-efficacy suggests risk-aware engagement, although the cross-sectional, self-reported findings do not establish causal or clinical effects. The focus on medical clerkship students and ward-based infection-prevention practice extends research that has largely examined GenAI adoption, acceptance, academic support, learning engagement, or clinical reasoning [2,4,6,9,64]. Medical schools and teaching hospitals should therefore treat GenAI as a supervised learning support, with patient-data protection, protocol alignment, verification of AI-generated outputs, and supervisor-led reflection incorporated into clerkship guidance. Perceived control over AI interaction should not be interpreted as evidence of clinical oversight or accountability. Future longitudinal, interventional, and multisite studies should assess observed behavior and supervisor-rated performance.

## Data Availability

The data generated and analyzed during this study are not publicly available because they contain participant responses from medical clerkship students and were collected under institutional ethics approval with confidentiality safeguards. Deidentified data may be made available from the corresponding author upon reasonable request and subject to ethics approval and institutional data-sharing requirements.

## Appendix

Multimedia Appendix 1 The conceptual framework of the hypothesized associations among generative AI–related perceptions, self-efficacy in AI-generated usage, perceived AI self-control balance, promotion of patient safety, and self-reported ward-based infection-prevention practice.

Multimedia Appendix 2 Participant flow through the sequential exploratory mixed methods study. Eight students completed the qualitative phase, 238 responses were received in the quantitative phase, and 214 responses were included in the final partial least squares structural equation modeling analysis.

Multimedia Appendix 3 Definitions and response scales for the 8 constructs included in the quantitative model.

Multimedia Appendix 4 Measurement items, outer loadings, descriptive statistics, internal consistency estimates, and average variance extracted for the eight constructs examined in the partial least squares structural equation modeling analysis.

Multimedia Appendix 5 Each cell reports the heterotrait-monotrait ratio of correlations point estimate followed by its bootstrapped 95% CI. None of the CIs included 1.00. Upper confidence limits exceeded 0.90 for AI-trust calibration literacy (AI-TCL)–AI-perceived algorithmic fairness and reached 0.909 for AI-TCL–AI-privacy concern toward AI monitoring and NIFRDT–AI-explainability quality exposure; these pairs are discussed as borderline in the Results.

Multimedia Appendix 6 Partial least squares structural equation modeling measurement model of generative AI–related factors, human-centered mediators, promotion of patient safety, and ward-based infection-prevention practice. Values beside the indicators are outer loadings, and values within the constructs are Cronbach α coefficients.

Multimedia Appendix 7 Structural model of generative AI–related factors, human-centered mediators, promotion of patient safety, and ward-based infection-prevention practice among medical clerkship students in Jakarta Metropolitan Area, Indonesia.

## Abbreviations

AVE: average variance extracted
BC: bias-corrected
CVPAT: Cross-Validated Predictive Ability Test
GenAI: generative AI
HAI: health care–associated infection
HTMT: heterotrait-monotrait ratio of correlations
JARS: Journal Article Reporting Standards
IPMA: importance-performance map analysis
MCAR: missing completely at random
NFI: normed fit index
PAISCB: perceived AI self-control balance
PLS-SEM: partial least squares structural equation modeling
SOAP: subjective-objective-assessment-plan
SRMR: standardized root mean square residual
TAM: technology acceptance model
