# Supplementary material for: Generative AI Factors, Cognitive Engagement, and Patient Safety Learning in Medical Education: Mixed Methods Study
Source: JMIR Med Educ. 2026 Oct 27;12:e100828. doi: 10.2196/100828 (PMC13752588; doi:10.2196/100828)
Supplement: Supplementary file 1 [file mededu_v12i1e100828_app3.docx]

| **Constructs** | **Conceptualized Definition** | **Response Scale** |
| --- | --- | --- |
| AI-Perceived Algorithmic Fairness | A subjective user’s judgement of fairness by transparency, accountability, and legitimacy of algorithmic decisions.[25] | 5-point Likert |
| AI-Trust Calibration Literacy | Human user’s understanding and skills to recognize when trust to artificial intelligence agents is misaligned and adjust it with the actual reliability of the agents.[54,77] | 7-point Likert |
| AI-Explainability Quality Exposure | The ability of a human user to understand the output of artificial intelligence agent by providing information about its internal logic, decision making process and factors influencing its output.[30,78] | 5-point Likert |
| AI-Privacy Concern Toward AI Monitoring | The anxiety or unease individuals feel regarding the collection, processing, and potential misuse of their personal information by AI systems, particularly when monitoring is continuous, covert, or involves sensitive data.[33,34] | 5-point Likert |
| Perceived AI Self-Control Balance | Students' perceived autonomy and interaction control during GenAI use, including control over prompting, interaction timing, prompt sequencing, and perceived adaptation of AI-supported learning activities. In this study, "balance" denotes students' perception of retaining control while learning with AI, not a division of authority between the learner and the system, and does not represent clinical oversight or accountable decision-making.[38] | 6-point Likert |
| Self-Efficacy in AI Generated Usage | An individual’s general belief in their ability to effectively use and interact with AI technologies or products, encompassing perceived assistance, anthropomorphic interaction, comfort with AI, and technological skills.[35,36] | 5-point Likert |
| Promotion of Patient Safety | A Medical Clerkship Student responsibility to provide care that minimizes risks and harm to service users. It avoids injuries to patients from the care that is intended to help them. [42] | 5-point Likert |
| Ward-Based Infection-Prevention Practice | The consistent implementation of multimodal infection prevention and control (IPC) interventions within hospital wards to reduce healthcare-associated infection (HAI) transmission and incidence among inpatients.[79] | 5-point Likert |

References

25. Yang Q, Lee Y-C. Ethical AI in Financial Inclusion: The Role of Algorithmic Fairness on User Satisfaction and Recommendation. Big Data Cogn Comput Multidisciplinary Digital Publishing Institute; 2024 Sept;8(9):105. doi: 10.3390/bdcc8090105

30. Holzinger A, Carrington A, Müller H. Measuring the Quality of Explanations: The System Causability Scale (SCS). KI - Künstl Intell 2020 June 1;34(2):193-198. doi: 10.1007/s13218-020-00636-z

33. Menard P, Bott GJ. Artificial intelligence misuse and concern for information privacy: New construct validation and future directions. Inf Syst J 2025;35(1):322?367. doi: 10.1111/isj.12544

34. Schepman A, Rodway P. Initial validation of the general attitudes towards Artificial Intelligence Scale. Comput Hum Behav Rep 2020 Jan 1;1:100014. doi: 10.1016/j.chbr.2020.100014

35. Wang Y-Y, Chuang Y-W. Artificial intelligence self-efficacy: Scale development and validation. Educ Inf Technol 2024 Mar 1;29(4):4785-4808. doi: 10.1007/s10639-023-12015-w

36. Carolus A, Koch MJ, Straka S, Latoschik ME, Wienrich C. MAILS - Meta AI literacy scale: Development and testing of an AI literacy questionnaire based on well-founded competency models and psychological change- and meta-competencies. Comput Hum Behav Artif Hum 2023 Aug 1;1(2):100014. doi: 10.1016/j.chbah.2023.100014

38. Wang F, Cheung ACK, Chai CS, Liu J. Development and validation of the perceived interactivity of learner-AI interaction scale. Educ Inf Technol 2025 Mar;30(4):4607-4638. doi: 10.1007/s10639-024-12963-x

42. Oldland E, Hutchinson AM, Redley B, Mohebbi M, Botti M. Evaluation of the validity and reliability of the Nurses? Responsibility in Healthcare Quality Questionnaire: An instrument design study. Nurs Health Sci 2021;23(2):525-537. doi: 10.1111/nhs.12844

54. Sidra S, Mason C. Generative AI in Human-AI Collaboration: Validation of the Collaborative AI Literacy and Collaborative AI Metacognition Scales for Effective Use. Int J Human-Computer Interact Taylor & Francis; 2025 Aug 19;0(0):1-25. doi: 10.1080/10447318.2025.2543997

77. Okamura K, Yamada S. Adaptive trust calibration for human-AI collaboration. Lv C, editor. PLOS ONE 2020 Feb 21;15(2):e0229132. doi: 10.1371/journal.pone.0229132

78. Hamida SU, Chowdhury MJM, Chakraborty NR, Biswas K, Sami SK. Exploring the Landscape of Explainable Artificial Intelligence (XAI): A Systematic Review of Techniques and Applications. Big Data Cogn Comput Multidisciplinary Digital Publishing Institute; 2024 Nov;8(11):149. doi: 10.3390/bdcc8110149

79. Price L, Gozdzielewska L, Hendry K, McFarland A, Reilly J. Effectiveness of national and subnational interventions for prevention and control of health-care-associated infections in acute hospitals in high-income and upper-middle-income counties: a systematic review update. Lancet Infect Dis Elsevier; 2023 Sept 1;23(9):e347-e360. PMID:37023784
