# Supplementary material for: Generative AI Factors, Cognitive Engagement, and Patient Safety Learning in Medical Education: Mixed Methods Study
Source: JMIR Med Educ. 2026 Oct 27;12:e100828. doi: 10.2196/100828 (PMC13752588; doi:10.2196/100828)
Supplement: Supplementary file 2 [file mededu_v12i1e100828_app4.docx]

| **Construct** | **Codes** | **Indicators** | **Outer Loadings** |
| --- | --- | --- | --- |
| AI-perceived algorithmic fairness[25] | AI-PAF1 | I feel Generative AI treats users equally without discrimination based on socioeconomic status. | 0.746 |
|  | AI-PAF2 | I feel that AI-based services use objective data and are free from framing (algorithms that favor certain interests). | 0.828 |
|  | AI-PAF3 | I feel that the decisions or recommendations generated by AI-based services are fair and do not favor any group. | 0.731 |
|  | AI-PAF4 | I feel that AI-based services do not provide preferential treatment to any group based on certain interests. | 0.733 |
|  |  | Mean = 4.109, CA = 0.758, rho_a = 0.761, rho_c = 0.846, AVE = 0.579 |  |
| AI-trust calibration literacy[54] | AI-TCL1 | I review the conclusions I draw from AI to ensure I don't accept or reject the recommendations too quickly. | 0.711 |
|  | AI-TCL2 | When using AI, I strive to be consistent with my initial goals when prompting or assigning tasks to the AI to ensure I achieve the results I need. | 0.810 |
|  | AI-TCL3 | When using AI, I try not to be influenced by the desire for quick results (mental shortcuts) without further analyzing the information provided by the AI. | 0.765 |
|  | AI-TCL4 | I recognize that my ability to prompt AI is still limited, so I cannot accept the recommendations from AI at face value. | 0.791 |
|  |  | Mean = 3.994, CA = 0.771, rho_a = 0.779, rho_c = 0.853, AVE = 0.593 |  |
| AI-explainability quality exposure[30] | AI-EQE1 | I easily understood the explanations provided by the AI in the context of my needs in carrying out my duties as a Medical Clerkship Student. | 0.803 |
|  | AI-EQE2 | I was able to increase the detail of the explanations through more specific prompts based on my needs. | 0.866 |
|  | AI-EQE3 | I felt that the AI's explanations helped me understand the cause-and-effect relationships (e.g., the causes of nosocomial infections) of the problems I raised through the AI's prompts. | 0.894 |
|  | AI-EQE4 | I was able to use these explanations in my activities as a Medical Clerkship Student in caring patients on the ward. | 0.892 |
|  |  | Mean = 4.134, CA = 0.887, rho_a = 0.888, rho_c = 0.922, AVE = 0.748 |  |
| AI-privacy concern toward AI monitoring[34] | AI-PCAM1 | I feel that AI can covertly collect personal information about me. | 0.911 |
|  | AI-PCAM2 | Sometimes I think AI can identify data about diseases in certain areas without realizing it. | 0.906 |
|  | AI-PCAM3 | I feel that there is a risk that my personal data obtained by AI could be misused without my permission. | 0.877 |
|  |  | Mean = 4.079, CA = 0.880, rho_a = 0.885, rho_c = 0.926, AVE = 0.806 |  |
| Self-efficacy in AI generated usage[36] | SEAIGU1 | In tight scheduling situations, I can rely on my ability to use AI to assist me with my work as a Medical Clerkship Student in a Hospital. | 0.844 |
|  | SEAIGU2 | I can choose the most appropriate prompting technique for my needs. | 0.905 |
|  | SEAIGU3 | I know how to validate the information I receive from AI to complete my medical tasks. | 0.910 |
|  | SEAIGU4 | I am familiar with the specific services provided by AI (e.g., creating images, diagrams, or analyzing numerical data in AI). | 0.913 |
|  | SEAIGU5 | I can stay up-to-date on the latest AI service features that meet my needs as a Medical Clerkship Student. | 0.543 |
|  |  | Mean = 4.186, CA = 0.882, rho_a = 0.896, rho_c = 0.918, AVE = 0.698 |  |
| Perceived AI self-control balance[38] | PAISCB1 | When using AI, I still feel free to choose what I want to do regardless of the AI's recommendations. | 0.776 |
|  | PAISCB2 | When I use AI, my prompting capabilities determine the learning experience I will have. | 0.799 |
|  | PAISCB3 | When using AI, I have complete control over when to start and end my sequel prompting. | 0.727 |
|  | PAISCB4 | When using AI, I am free to determine the order in which I deliver prompts to meet my goals. | 0.790 |
|  | PAISCB5 | I feel that AI can adapt itself to my preferences. | 0.838 |
|  |  | Mean = 3.662, CA = 0.846, rho_a = 0.847, rho_c = 0.890, AVE = 0.619 |  |
| Promotion of patient safety[42] | PPS1** | I anticipate high-risk situations on the rotation unit I work on. | 0.912 |
|  | PPS2** | I understand the role of environmental factors such as workflow, ergonomics, and resources in impacting patient safety. | 0.822 |
|  | PPS3** | I support standardized systems that reduce reliance on memory (e.g., handover devices, electronic medication records) for tasks assigned to Medical Clerkship Student. | 0.857 |
|  |  | Mean = 4.127, CA = 0.841, rho_a = 0.972, rho_c = 0.899, AVE = 0.748 |  |
| Ward-based infection-prevention practice [41] | NIFRDT1* | Not washing hands before contact with other patients. | 0.752 |
|  | NIFRDT2* | Wearing rings, bracelets, or watches. | 0.743 |
|  | NIFRDT3* | Not changing personal protective equipment (such as gloves) between patients. | 0.747 |
|  | NIFRDT4* | Not wearing gloves when in contact with body fluids and blood. | 0.769 |
|  |  | Mean = 3.924, CA = 0.747, rho_a = 0.749, rho_c = 0.840, AVE = 0.567 |  |

CA = Cronbach’s Alpha, rho_c = Composite Reliability, AVE = Average Variance Extracted

*Note. NIFRDT indicators were reverse-coded before PLS-SEM analysis; therefore, higher scores indicate better self-reported ward-based infection-prevention practice.

** Note. For promotion of patient safety, the rho_A estimate was unstable across bootstrap resamples and exceeded composite reliability; internal consistency for this construct was therefore evaluated from Cronbach's alpha and composite reliability.

References

25. Yang Q, Lee Y-C. Ethical AI in Financial Inclusion: The Role of Algorithmic Fairness on User Satisfaction and Recommendation. Big Data Cogn Comput Multidisciplinary Digital Publishing Institute; 2024 Sept;8(9):105. doi: 10.3390/bdcc8090105

30. Holzinger A, Carrington A, Müller H. Measuring the Quality of Explanations: The System Causability Scale (SCS). KI - Künstl Intell 2020 June 1;34(2):193-198. doi: 10.1007/s13218-020-00636-z

34. Schepman A, Rodway P. Initial validation of the general attitudes towards Artificial Intelligence Scale. Comput Hum Behav Rep 2020 Jan 1;1:100014. doi: 10.1016/j.chbr.2020.100014

36. Carolus A, Koch MJ, Straka S, Latoschik ME, Wienrich C. MAILS - Meta AI literacy scale: Development and testing of an AI literacy questionnaire based on well-founded competency models and psychological change- and meta-competencies. Comput Hum Behav Artif Hum 2023 Aug 1;1(2):100014. doi: 10.1016/j.chbah.2023.100014

38. Wang F, Cheung ACK, Chai CS, Liu J. Development and validation of the perceived interactivity of learner-AI interaction scale. Educ Inf Technol 2025 Mar;30(4):4607-4638. doi: 10.1007/s10639-024-12963-x

41. Aktas E, Çövener ÖzçeliK Ç, Ocakçi AF. Reliability and Validity of Nurses? Experiences of Infection Prevention and Control Questionnaire. Clin Exp Health Sci 2020 June 29;10(2):137-141. doi: 10.33808/clinexphealthsci.646357

42. Oldland E, Hutchinson AM, Redley B, Mohebbi M, Botti M. Evaluation of the validity and reliability of the Nurses? Responsibility in Healthcare Quality Questionnaire: An instrument design study. Nurs Health Sci 2021;23(2):525-537. doi: 10.1111/nhs.12844

54. Sidra S, Mason C. Generative AI in Human-AI Collaboration: Validation of the Collaborative AI Literacy and Collaborative AI Metacognition Scales for Effective Use. Int J Human-Computer Interact Taylor & Francis; 2025 Aug 19;0(0):1-25. doi: 10.1080/10447318.2025.2543997
