# Supplementary material for: Generative AI Factors, Cognitive Engagement, and Patient Safety Learning in Medical Education: Mixed Methods Study
Source: JMIR Med Educ. 2026 Oct 27;12:e100828. doi: 10.2196/100828 (PMC13752588; doi:10.2196/100828)
Supplement: Supplementary file 3 [file mededu_v12i1e100828_app5.docx]

|  | AI-EQE | AI-PAF | AI-PCAM | AI-TCL | NIFRDT | PAISCB | PPS | SEAIGU |
| --- | --- | --- | --- | --- | --- | --- | --- | --- |
| AI-EQE |  |  |  |  |  |  |  |  |
| AI-PAF | 0.670  CI (0.557; 0.766) |  |  |  |  |  |  |  |
| AI-PCAM | 0.742  CI (0.626; 0.829) | 0.620  CI (0.493; 0.719) |  |  |  |  |  |  |
| AI-TCL | 0.811  CI (0.711; 0.883) | 0.861  CI (0.765; 0.940) | 0.830  CI (0.736; 0.909) |  |  |  |  |  |
| NIFRDT | 0.845  CI (0.770; 0.909) | 0.609  CI (0.485; 0.709) | 0.712  CI (0.596; 0.804) | 0.776  CI (0.665; 0.866) |  |  |  |  |
| PAISCB | 0.627 CI (0.502; 0.730) | 0.556 CI (0.431; 0.659) | 0.450  CI (0.289; 0.593) | 0.552  CI (0.418; 0.661) | 0.698  CI (0.558; 0.807) |  |  |  |
| PPS | 0.363  CI (0.208; 0.510) | 0.509  CI (0.370; 0.634) | 0.416  CI (0.266; 0.545) | 0.497  CI (0.357; 0.619) | 0.216  CI (0.103; 0.349) | 0.116  CI (0.051; 0.141) |  |  |
| SEAIGU | 0.778  CI (0.685; 0.855) | 0.745  CI (0.637; 0.828) | 0.776  CI (0.691; 0.844) | 0.823  CI (0.742; 0.888) | 0.758  CI (0.640; 0.851) | 0.453  CI (0.303; 0.579) | 0.495  CI (0.370; 0.604) |  |

Note. HTMT=heterotrait-monotrait ratio of correlations; CI=confidence interval. Each cell reports the HTMT point estimate followed by its bootstrapped 95% CI. None of the confidence intervals included 1.00. Upper confidence limits exceeded 0.90 for AI-TCL–AI-PAF and reached 0.909 for AI-TCL–AI-PCAM and NIFRDT–AI-EQE; these pairs are discussed as borderline in the Results.
